# NKT cells promote both type 1 and type 2 inflammatory responses in a mouse model of liver fibrosis

**DOI:** 10.1038/s41598-020-78688-2

**Published:** 2020-12-11

**Authors:** Julia Nilsson, Maria Hörnberg, Anja Schmidt-Christensen, Kajsa Linde, Maria Nilsson, Marine Carlus, Saskia F. Erttmann, Sofia Mayans, Dan Holmberg

**Affiliations:** 1 Department of Experimental Medical Sciences, Lund University Diabetes Center, Clinical Research Center, Lund University, Jan Waldenströms gata 35, 214 28 Malmö, Sweden; 2 InfiCure Bio AB, Tvistevägen 48 C, 907 36 Umeå, Sweden; 3 Carlus Pathology Consulting, 2 rue de la Libération, 76630 Bellengreville, France; 4 Department of Molecular Biology, Umeå University, 901 87 Umeå, Sweden

**Keywords:** Inflammation, Liver diseases

## Abstract

Sterile liver inflammation and fibrosis are associated with many liver disorders of different etiologies. Both type 1 and type 2 inflammatory responses have been reported to contribute to liver pathology. However, the mechanisms controlling the balance between these responses are largely unknown. Natural killer T (NKT) cells can be activated to rapidly secrete cytokines and chemokines associated with both type 1 and type 2 inflammatory responses. As these proteins have been reported to accumulate in different types of sterile liver inflammation, we hypothesized that these cells may play a role in this pathological process. We have found that a transgenic NKT (tgNKT) cell population produced in the immunodeficient 2,4αβNOD.*Rag2*^−/−^ mice, but not in 2,4αβNOD.*Rag2*^+/−^ control mice, promoted a type 1 inflammatory response with engagement of the NOD-, LRR- and pyrin domain-containing protein-3 (NLRP3) inflammasome. The induction of the type 1 inflammatory response was followed by an altered cytokine profile of the tgNKT cell population with a biased production of anti-inflammatory/profibrotic cytokines and development of liver fibrosis. These findings illustrate how the plasticity of NKT cells modulates the inflammatory response, suggesting a key role for the NKT cell population in the control of sterile liver inflammation.

## Introduction

Chronic liver inflammation regardless of etiology can lead to liver fibrosis and, eventually, cirrhosis^1^. The underlying pathological processes may be viral or bacterial infection or sterile liver inflammation caused by alcoholic liver disease (ALD), nonalcoholic steatohepatitis (NASH), or autoimmune hepatitis (AIH). Irrespective of the etiology, the process is characterized by the presence of persistent liver injury and involves multiple cell populations, including Kupffer cells (KCs), liver tissue-resident macrophages, monocyte-derived macrophages and other recruited inflammatory cells^2,3^. In sterile liver inflammation, the NOD-, LRR- and pyrin domain-containing protein-3 (NLRP3) inflammasome has been suggested to play a central role^4^. Activation of the NLRP3 inflammasome is a two-step process that includes an initial priming step involving the engagement of Toll-like receptors (TLRs) or the binding of different cytokines, such as tumor necrosis factor α (TNFα) or interleukin-1β (IL-1β), to their respective receptors. This receptor activates nuclear factor kappa B (NF-κB), resulting in the production of pro-IL-1β and pro-IL-18 proteins^5–7^. A second signal is required to initiate the assembly and subsequent activation of the NLRP3 inflammasome complex. This activation is triggered by not only different molecular and cellular events, such as mitochondrial dysfunction and/or ionic flux, but also in response to a large variety of molecules, e.g. those released from damaged liver cells, including adenosine triphosphate (ATP). Inflammasome activation results in the caspase-1-mediated maturation of pro-IL-1β and pro-IL-18 into their biologically active forms.

Type 1 inflammatory responses have been generally associated with the initiation of sterile liver inflammation, including NAFLD^8–10^. An initial phase of recruitment and activation of innate immune cells that includes the production of pro-inflammatory cytokines and chemokines, such as IL-6, TNFα and CC motif chemokine ligand 2 (CCL2), drives the type 1 inflammatory response that contributes to local tissue destruction. This pro-inflammatory response also triggers the immune system to prepare for a transition to repair mode, in which key inflammatory cells such as KCs and monocyte-derived macrophages switch from a type 1 pro-inflammatory to a type 2 anti-inflammatory/reparative response^9,11^. IL-33^12^ may constitute a key molecule in this process by serving as an alarmin activating type 2 innate lymphoid cell (ILC2) and contributing to the activation of hepatic stellate cells (HSCs)^13^. During the inflammatory response, IL-33 expression is induced in a variety of cell types, including epithelial and endothelial cells^14^. However, in liver fibrosis, the main source of IL-33 has been reported to be stressed hepatocytes^13,15^. The reparative type 2 response involves a plethora of inflammatory cells, including ILC2s, M2 macrophages, eosinophils and natural killer T (NKT) cells^16^. A major component of the type 2 inflammatory response is transforming growth factor β1 (TGF-β1), which can activate HSCs, the most potent fibrogenic cells and mediators of the secretion and deposition of extracellular matrix (ECM)^16–19^.

While type 1 and type 2 inflammatory reactions have been reported in various chronic liver inflammatory and fibrotic conditions, including NASH^8,9^, the mechanisms controlling the balance between pro-inflammatory and reparative responses are not well understood^16^. Accumulating data have suggested that NKT cells, naturally homing to the liver, are associated with chronic liver inflammation and fibrosis^20–22^. NKT cells have been reported to activate the NLRP3 inflammasome^23–25^ and to produce pro-inflammatory as well as anti-inflammatory cytokines that drive tissue repair and fibrosis^20^. Two subsets of CD1d-restricted NKT cells have been identified in both mice and humans: invariant NKT (iNKT) and type 2 NKT cells^26–29^. These subsets play important roles in the regulation of liver inflammation, where they influence other inflammatory cells through their innate-like property of rapid secretion of both pro-inflammatory and anti-inflammatory cytokines. iNKT^21,30,31^ and type 2 NKT^32^ cells have been described as having both immunostimulatory and regulatory properties that enable their roles as mediators of inflammatory responses in many diseases.

We have previously described a new model for liver fibrosis, the nonobese diabetic inflammation and fibrosis (NIF) mouse^33,34^, in which a transgenic NKT cell population induces chronic inflammation and fibrosis. Here, we demonstrate that, in this model, transgenic NKT cells drive a type 1 inflammatory response through the production of pro-inflammatory cytokines involving the activation of the NLRP3 inflammasome and promote the switch to a predominantly anti-inflammatory, reparative/profibrotic response through the production of type 2 cytokines such as IL-13. Together, these findings demonstrate that NKT cells, through the plasticity of their cytokine expression profile, can play important roles in the control of chronic liver inflammation.

## Results

### The NIF mice develop chronic liver inflammation

**Figure 1:**
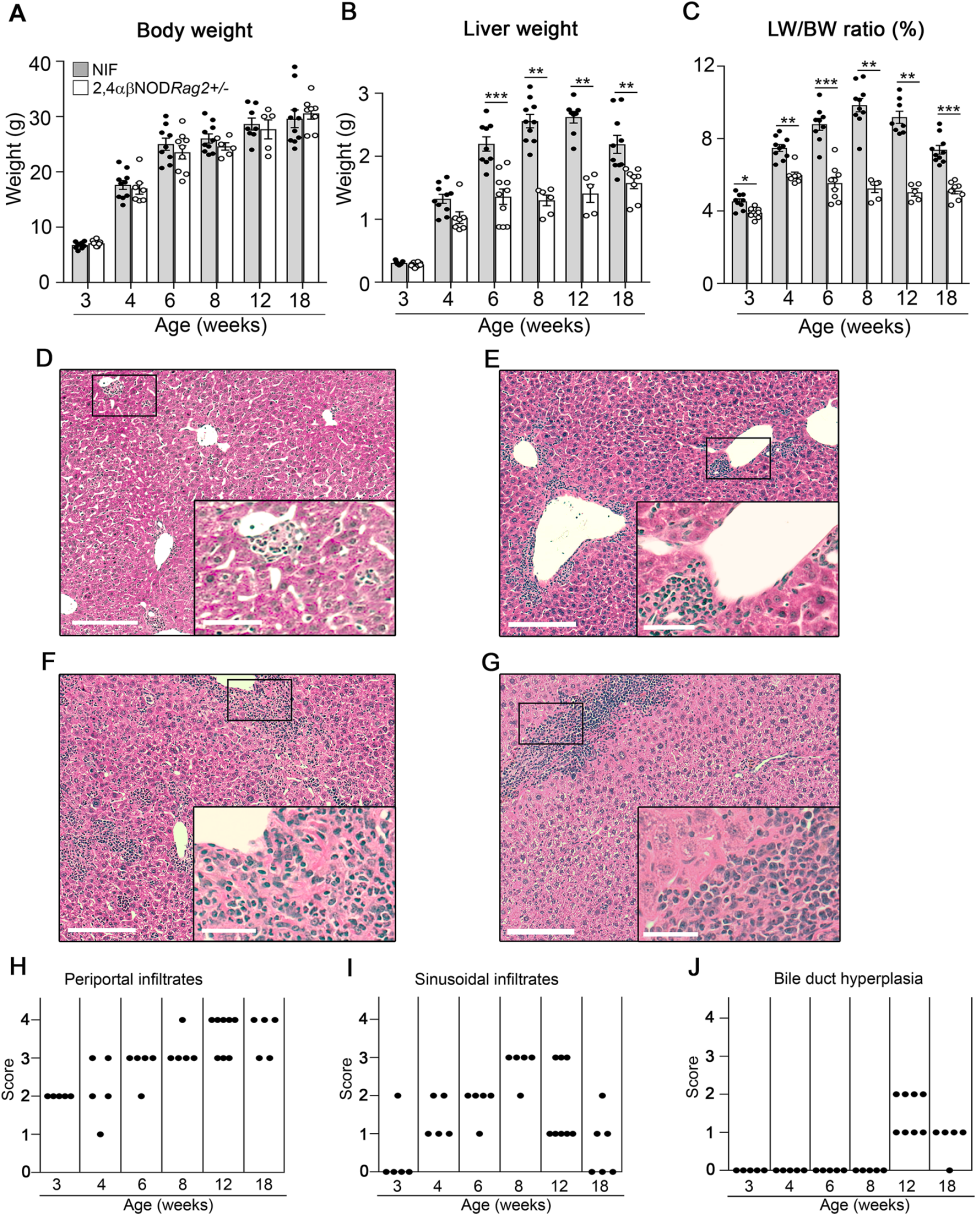
Kinetics of liver inflammation in the NIF mice. (**A**) Body weights, (**B**) liver weights and (**C**) liver-to-body weight ratios of the NIF mice (filled circle, shaded bars) (n = 8–11) and 2,4αβNOD.*Rag*2^+/−^ control mice (open circle, open bars) (n = 5–10). *P < 0.05, **P < 0.01 and ***P < 0.001, Mann–Whitney U test after Bonferroni correction. Representative H&E-stained liver sections from (**D**) 4-week-old (**E**) 6-week-old (**F**) 8-week-old and (**G**) 18-week-old NIF mice. Framed areas are highlighted. Scale bars are 200 μm in the overview images and 50 μm in the highlighted images. Semiquantitative assessment of (**H**) periportal infiltrates, (**I**) sinusoidal infiltrates and (**J**) bile duct hyperplasia in the NIF mice (n = 8–11) (n = 5–10) at the indicated ages.

NIF mice spontaneously developed chronic liver inflammation and fibrosis initiated by a transgenic population of NKT cells while 2,4αβNOD.Rag2^+/−^ littermate control mice did not^34^. To gain further insight into the mechanisms underlying this process, we followed the kinetics according to the liver phenotype in mice from 3 to 18 weeks of age. As illustrated in Fig. 1A–C, hepatomegaly developed at 4 weeks of age with the peak liver weight (Fig. 1B) and liver-to-body weight ratio (Fig. 1C) reached in mice approximately 8–12 weeks of age. As expected, the males gained more weight than the females did, but no gender differences in the liver-to-body weight ratio was observed (Fig. S1). Hematoxylin and eosin (H&E) staining of liver sections revealed an accumulation of inflammatory cells in the NIF mouse livers (Fig. 1D–J). Therefore, at 3–4 weeks of age, all the mice displayed slight periportal infiltrates composed of mixed inflammatory cells (Fig. 1D,H). At 4 weeks of age, all the animals had minimal to moderate periportal and minimal to slight sinusoidal mixed inflammatory cell infiltrates (Fig. 1E,H,I). At 6 weeks of age, similar lesions were observed, but the severity was increased. This injury development was associated with few foci of degeneration/necrosis in three of five mice (Fig. 1F). The severity of these lesions was increased in the 8- and 12-week-old NIF mice, after which the progression halted (Fig. 1G). Quantification by scoring the periportal (Fig. 1H) and sinusoidal (Fig. 1I) inflammatory infiltrates and degree of bile duct hyperplasia (Fig. 1J and Fig. S2) confirmed the progression of liver inflammation.

Despite the progressive inflammation and evidence of necrosis in the liver of the NIF mice, we observed only minor alterations in serum alanine aminotransferase (ALT) and aspartate aminotransferase (AST) (Fig. S3). While no alterations in serum triglycerides were observed, the serum levels of bile acids were moderately elevated in the NIF mice.

### NKT cells drive an initial type 1 inflammatory response in the livers of the NIF mice

**Figure 2:**
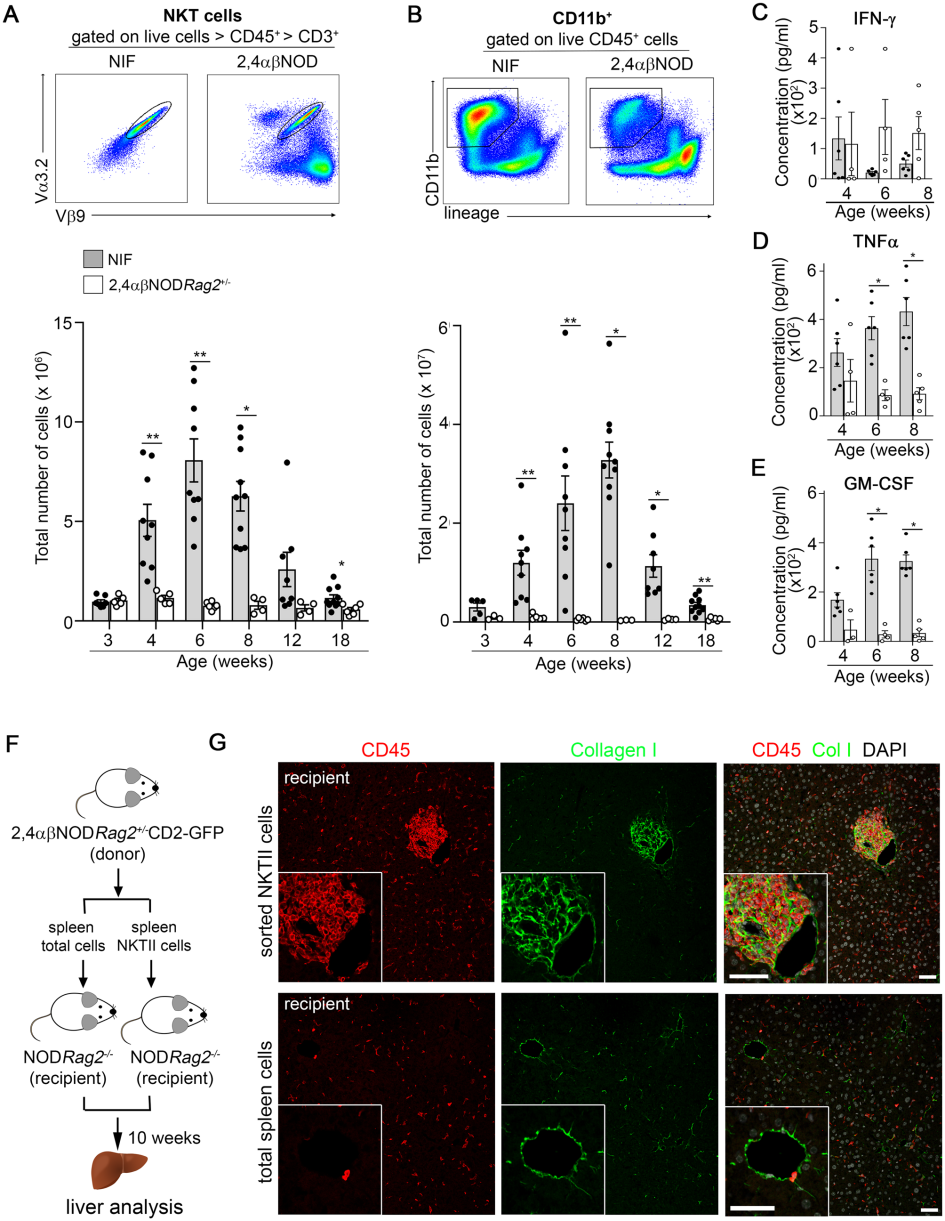
The accumulation of NKT cells precedes the recruitment of CD11b^+^ myeloid cells and correlates with a type 1 inflammatory response in the livers of the NIF mice. Single cell suspensions from livers of the NIF and 2,4αβNOD.*Rag*2^+/−^ mice were analyzed by flow cytometry. (**A**) Total number of Vβ9^+^Vα3.2^+^ NKT cells gated from CD45^+^ viable cells. NIF mice (filled circle, shaded bars) (n = 7–10) and 2,4αβNOD.*Rag*2^+/−^ control mice (open circle, open bars) (n = 4–6). (**B**) Total number of CD11b^+^ cells gated from viable cells. NIF mice (filled circle, shaded bars) (n = 5–10) and 2,4αβNOD.*Rag*2^+/−^ control mice (open circle, open bars) (n = 3–6) (details of the full gating strategy are presented in Fig. S4). Levels of (**C**) IFN-γ, (D) TNFα, and (**E**) GM-CSF in the supernatants of liver leukocytes stimulated for 24 h with anti-CD3 (4 µg/ml). NIF mice (filled circle, shaded bars) (n = 6) and 2,4αβNOD.*Rag*2^+/−^ control mice (open circle, open bars) (n = 3–5). Data are pooled from two independent experiments. *P < 0.05, **P < 0.01, and ***P < 0.001, Mann–Whitney U test after Bonferroni correction. A Wilcoxon ranked test was applied to compare the accumulation of CD11b^+^ leukocytes and NKT cells in mice 6 and 8 weeks old. (**F**) Outline of experimental design of adoptive transfer of total spleen cells or sorted NKT cells from 2,4αβNOD.*Rag*2^+/−^ mice to naïve NOD.*Rag2*^−/−^ recipient mice. (**G**). Immunohistochemical staining of representative cryosections from NOD.*Rag2*^−/−^ recipient mice sacrificed 10 weeks after receiving sorted NKT cells (upper row) or total spleen cells (lower row) stained with anti-CD45 (red), anti-collagen I (green) or DAPI.

We next used FACS analysis to study the kinetics of the accumulated inflammatory cells and specifically the NKT cells in the livers of the NIF and 2,4αβNOD.*Rag*2^+/−^ control mice (Fig. 2) (for the full gating strategy, see Fig. S4). We found that the NKT cells started accumulating in the liver of the NIF mice at 4 weeks of age, reaching a peak value at 6 weeks of age (Fig. 2A). However, the accumulation of CD11b^+^ leukocytes was significantly delayed and reached its maximum approximately 2 weeks after the NKT cell level peaked (Fig. 2B). (Overall cellular composition in NIF liver and spleen is provided in Fig. S5). Consistent with our previous report, the NKT cells in both the NIF and 2,4αβNOD.*Rag*2^+/−^ control mice expressed interferon γ (IFN-γ) and TNFα^34^, and we found that the liver leukocytes isolated from both the NIF and 2,4αβNOD.*Rag*2^+/−^ control mice expressed these cytokines after being stimulated with anti-CD3 antibodies (Fig. 2C,D). The levels of IFN-γ were decreased in the NIF mice 6 weeks of age and older compared to those in the 2,4αβNOD.*Rag*2^+/−^ control mice, the expression levels of TNFα were increased. Further, significant levels of granulocyte-macrophage colony-stimulating factor (GM-CSF) were expressed in NIF mice, while 2,4αβNOD.*Rag*2^+/−^ control mice expressed low or undetectable levels of this cytokine throughout the time period monitored (Fig. 2E). These findings are in line with the notion that the transgenic NKT cell population plays a role in initiating the development of a type 1 inflammatory response in the liver of NIF mice.

To directly test if the transgenic NKT cells could mediate the observed development of inflammation and fibrosis, we first introduced the CD2-GFP reporter transgene^35^ onto the genome of the 2,4αβNOD.Rag2^−/−^ (NIF) mouse generating 2,4αβNOD.Rag2^+/−^.CD2-GFP mice (Fig. S6). Similar to the 2,4αβNOD.Rag2^+/−^ control mice the 2,4αβNOD.Rag2^+/−^.CD2-GFP did not develop liver inflammation or fibrosis while 2,4αβNOD.Rag2^−/−^.CD2-GFP did. We next adoptively transferred either total spleen cells or sorted NKT cells from 2,4αβNOD.Rag2^+/−^.CD2-GFP mice into naïve NOD.Rag2^−/−^ recipients and the mice were sacrificed 10 weeks after adoptive transfer. As illustrated in Fig. 2F, mice that received sorted NKT cells but not those that received total spleen cells were found to develop liver inflammation and fibrosis (Fig. 2G and Fig. S7).

### Activation of the NLRP3 inflammasome pathway drives type 1 inflammation in the livers of the NIF mice

**Figure 3:**
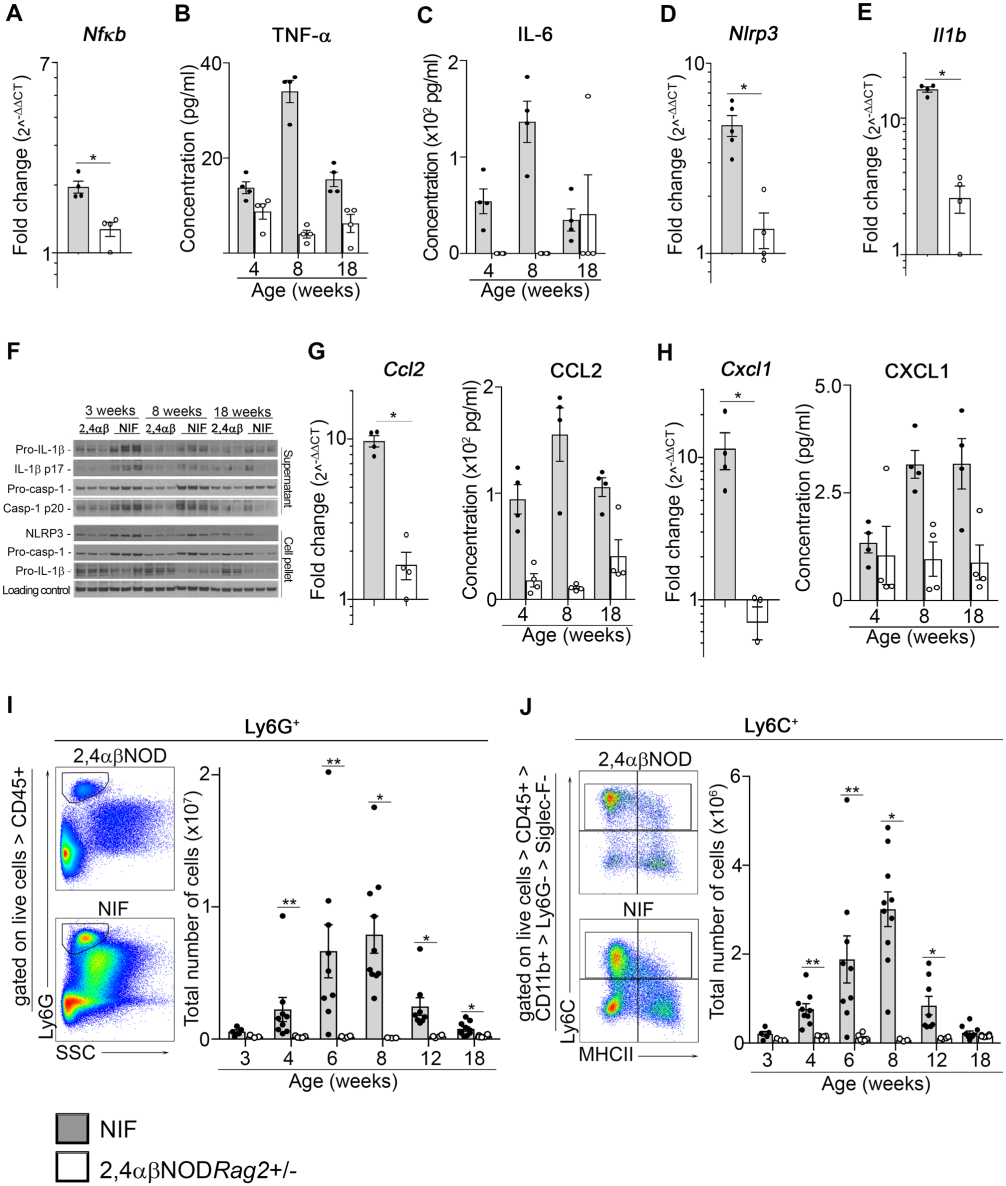
Liver inflammation is associated with NLRP3 inflammasome activation in the livers of the NIF mice. (**A**) RT-qPCR analysis of *Nfkb* gene expression in total liver lysates from the NIF mice (n = 4) and 2,4αβNOD.*Rag*2^+/−^ control mice (n = 4). Serum protein expression of (**B**) TNFα and (**C**) IL-6 in the NIF (n = 4) and 2,4αβNOD.*Rag*2^+/−^ control mice (n = 4). RT-qPCR analysis of (**D**) *Nlrp3* and (**E**) *Il1b* gene expression in the total liver lysates from the NIF mice (n = 5) and 2,4αβNOD.*Rag*2^+/−^ control mice (n = 4). (**F**) Immunoblots of IL-1β and processed Caspase-1 and NLRP3 in the livers from the NIF and 2,4αβNOD.*Rag*2^+/−^ control mice of the indicated ages (n = 3 per group). RT-qPCR analysis of the total liver lysates (left) and serum protein expression (right) of (**G**) CCL2 and (**H**) CXCL1 in the NIF (n = 4) and 2,4αβNOD.*Rag*2^+/−^ control mice (n = 4). Representative dot blot and statistics of the FACS analyses of single-cell suspensions prepared from the livers of the NIF and 2,4αβNOD.*Rag*2^+/−^ control mice gated: (**I**) Ly6G^+^ neutrophils and (**J**) Ly6C^+^ inflammatory monocytes. (Details of the full gating strategy are presented in Figure S4.) NIF mice (filled circle, shaded bars); 2,4αβNOD.*Rag*2^+/−^ control mice (open circle, open bars). * *P* < 0.05, ** *P* < 0.01, and *** *P* < 0.001, Mann–Whitney U test after Bonferroni correction.

The liver inflammation observed in the NIF mice at approximately 4 weeks of age and older was found to be associated with an increased expression of the *Nfkb* gene in the livers of the NIF mice (Fig. 3A). We also observed increased expression of the NFκB-regulated cytokines TNFα (Fig. 3B) and IL-6 (Fig. 3C); both of these type 1 pro-inflammatory cytokines reached a maximum level in mice at approximately 8 weeks of age, which corresponded to the time point at which maximum inflammation was observed in the livers of the NIF mice. Further, we noted that the expression of the *Nlrp3* and *Il1b* genes, known to be regulated by NFκB, was significantly increased in the NIF mouse livers (Fig. 3D,E, respectively). Because of the accumulating evidence that activation of the NLRP3 inflammasome pathway can play a critical role in sterile liver inflammation^4^, we next looked for the protein expression of inflammasome components NLRP3, IL-1β and caspase-1. Immunoblot analyses confirmed the increased NLRP3 and pro-IL-1β protein expression in the livers of the NIF mice compared to that in the 2,4αβNOD.*Rag*2^+/−^ control mice, as well as the increased expression of unprocessed pro-caspase-1 (Fig. 3F and Fig. S8). Since pro-caspase-1 is known to be constitutively expressed, we assumed that the increased expression of pro-caspase-1 was a consequence of the accumulation of inflammatory cells in the livers of the NIF mice.

In addition to the NFκB-mediated induction of NLRP3 and pro-IL-1β, full activation of the NLRP3 inflammasome requires additional signaling that leads to the assembly of the inflammasome complex, activation of caspase-1 and the subsequent production of biologically active IL-1β. To verify that full activation of the NLRP3 inflammasome occurred in the livers of the NIF mice, we used immunoblotting to analyze the cleavage of pro-caspase-1 and pro-IL-1β to caspase-1 and IL-1β, respectively. We found that the levels of processed caspase-1 and IL-1β (Fig. 3F) were increased in the livers of the NIF mice compared to those in the livers of the 2,4αβNOD.*Rag*2^+/−^ control mice. Thus, we concluded that the NKT cell-driven inflammation in the liver of NIF mice is at least partially induced by the activation of the NLRP3 inflammasome pathway.

In addition, we noted an increase in the neutrophil recruiting and activating chemokines CCL2 (Fig. 3G) and C-X-C motif ligand 1 (CXCL1) (Fig. 3H) both at the mRNA level in the livers and at the protein level in serum. Finally, and in accordance with these findings, we observed the recruitment of Ly6G^+^ neutrophils (Fig. 3I) and Ly6C^+^ monocytes (Fig. 3J) to the liver of the NIF mice; both of which are cellular components of type 1 inflammation^36,37^.

### Initial type 1 inflammation in the livers of the NIF mice is followed by the development of progressive liver fibrosis

**Figure 4:**
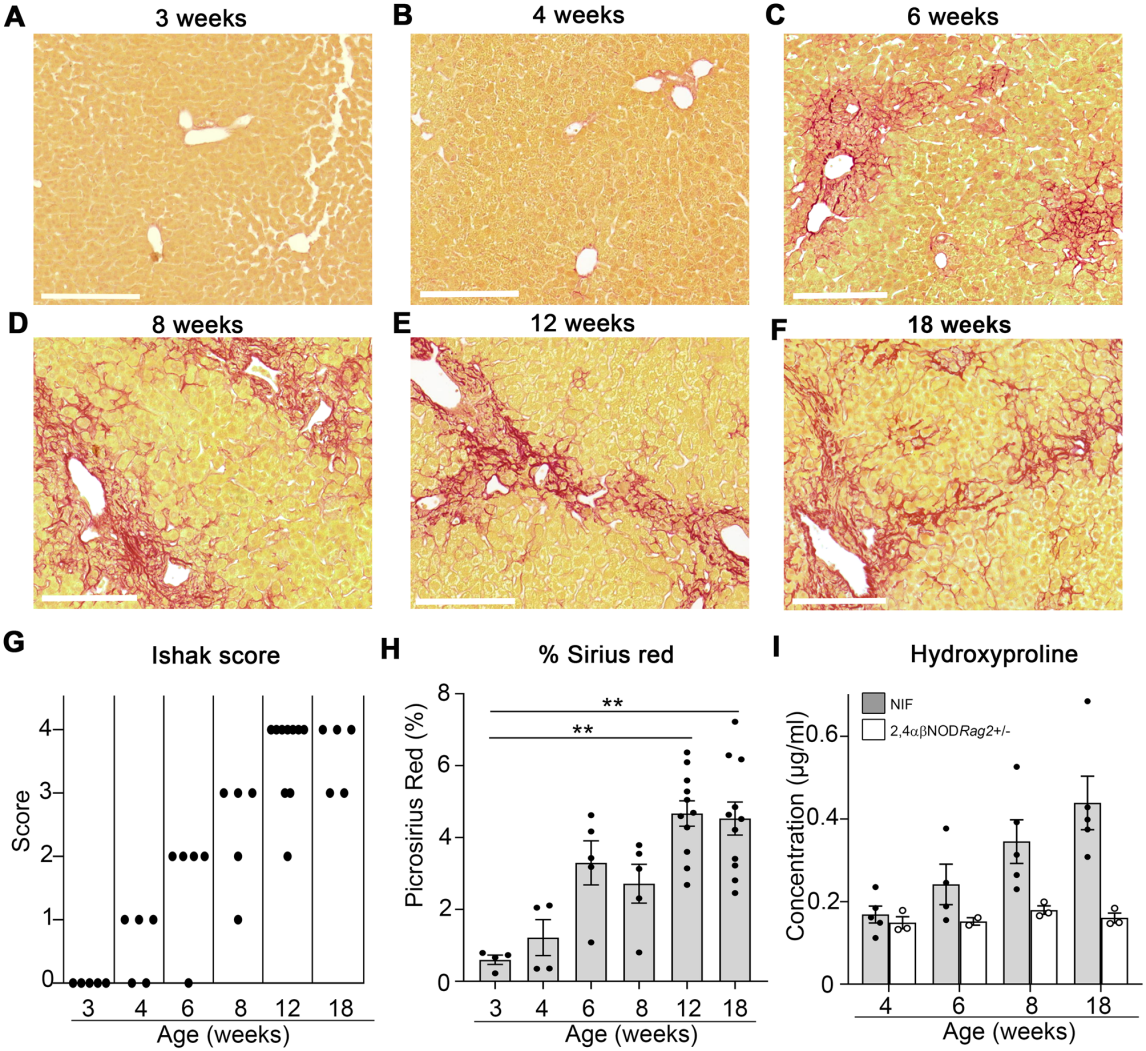
The initial type 1 inflammatory response in the livers of the NIF mice is followed by the development of progressive liver fibrosis. Representative picrosirius red (PSR)-stained liver sections from (**A**) 3-week-old (n = 5), (**B**) 4-week-old (n = 5), (**C**) 6-week-old (n = 5), (**D**) 8-week-old (n = 5), (**E**) 12-week-old (n = 11) and (**F**) 18-week-old (n = 5) NIF mice. Scale bars are 200 μm. (**G**) Ishak scores of the livers of the NIF mice at the indicated time points. (**H**) Quantification of PSR in the liver sections of the NIF mice at the indicated time points (see Fig. S9 for a more detailed description). (**I**) Hydroxyproline content in the liver tissue from the NIF mice (n = 4) (filled circle, shaded bars) and 2,4αβNOD.*Rag*2^+/−^ control mice (n = 3) (open circle, open bars). *P < 0.05, **P < 0.01, and ***P < 0.001, Mann–Whitney U test after Bonferroni correction.

The initial inflammatory reaction in the NIF mouse livers with an onset in mice of approximately 3–4 weeks of age was followed by the progressive development of liver fibrosis (Fig. 4). Microscopic examination of picrosirius red (PSR)-stained liver sections (Fig. 4A–F) revealed that the first signs of fibrosis appeared in the mice as young as 4 weeks old with few and small multifocal areas of degeneration/necrosis in three of the five animals and an Ishak score of 1 (Fig. 4G). Similar but more severe lesions were observed in mice 6 and 8 weeks old, with Ishak scores ranging from 1 to 3. In mice 12 weeks and older, moderate and marked periportal infiltrates were observed in all the animals and were associated with minimal to moderate levels of sinusoid infiltrates and minimal to moderate fibroplasia/fibrosis (Fig. 1H–J). At this time, minimal to moderate multifocal areas of degeneration/necrosis were noted in 50% of the animals, and Ishak scores were increased but never exceeded a score of 4 (Fig. 4E,G). The progression of the fibrosis, as determined by histopathology, was confirmed and quantitated by determining the levels of collagen accumulation with PSR staining (Fig. 4H). Moreover, the progression of fibrosis was reflected in the trend of increased hydroxyproline content levels in the liver tissue (Fig. 4I).

### The fibrogenesis of the NIF mouse liver depends on TGF-β signaling

**Figure 5:**
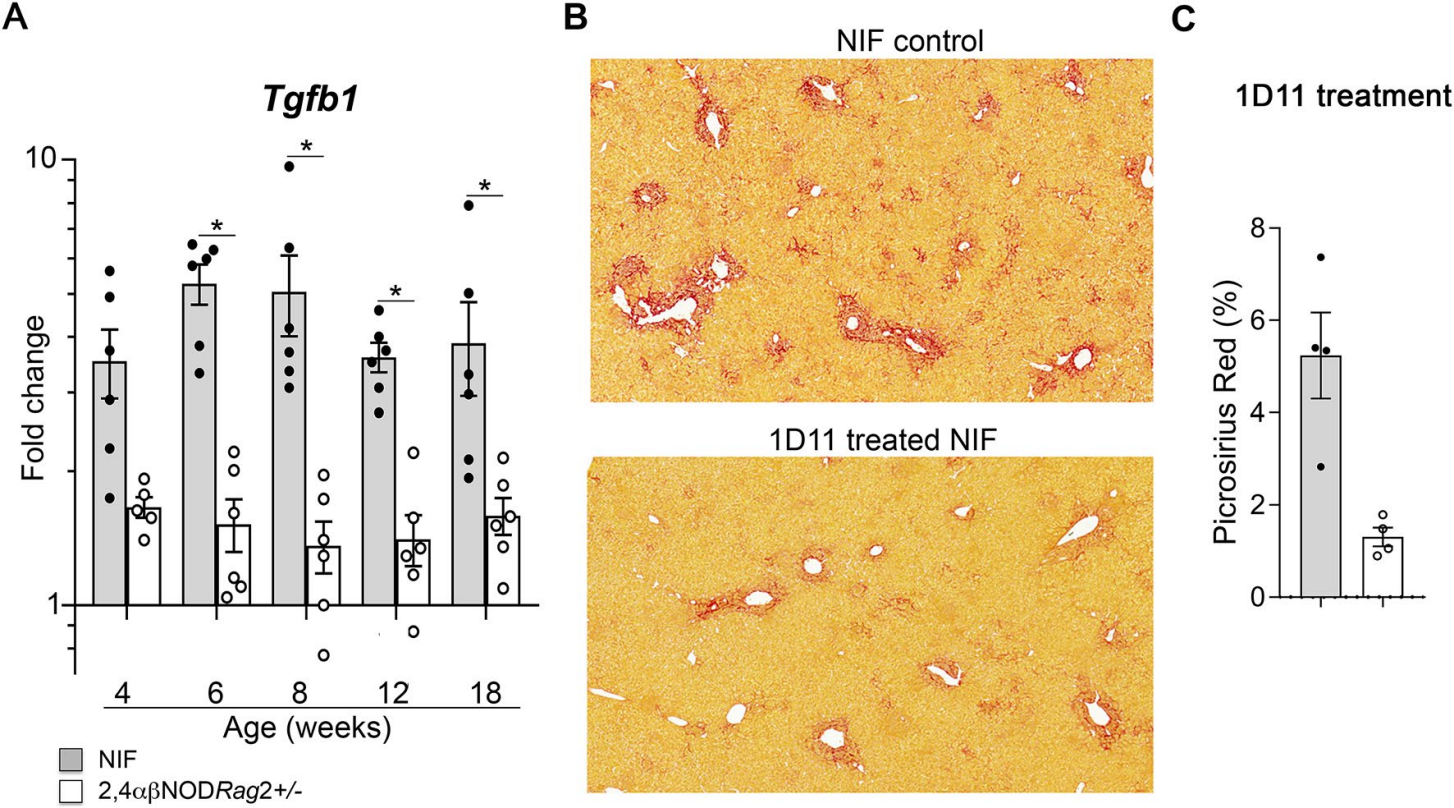
The development of fibrosis in the livers of the NIF mice is dependent on TGF-β1. (**A**) Real-time quantitative PCR (RT-qPCR) analysis of *Tgfb1* gene expression in the total liver lysates from the NIF mice (filled circle, shaded bars) (n = 5–6) and 2,4αβNOD.*Rag*2^+/−^ control mice (open circle, open bars) (n = 5–6). Data are pooled from three independent experiments. (**B**) Representative PSR-stained liver sections from 10-week-old NIF mice treated or not with anti-TGF-β1 antibody 1D11. Scale bars are 200 μm. (**C**) Quantification of PSR (Fig. S9 presents the details) in the liver sections from the NIF mice treated (filled circle, shaded bars) (n = 4) or not (open circle, open bars) (n = 5) for 4 weeks with anti-TGF-β1 antibody 1D11. *P < 0.05, **P < 0.01, and ***P < 0.001, Mann–Whitney U test after Bonferroni correction.

TGF-β1 has been identified as a major factor associated with the transition of chronic type 1 inflammation to a type 2 inflammatory response mediating repair and fibrogenic signals. To investigate its potential role in the observed development of liver fibrosis in the NIF mice, we analyzed the kinetics of the mRNA expression of the *Tgfb1* gene. RT-qPCR analyses revealed an increase in the expression of the *Tgfb1* gene in the NIF mice 4 weeks of age and older, and it remained elevated throughout the study compared to that in the 2,4αβNOD.*Rag*2^+/−^ control mice (Fig. 5A). To directly test the causative action of TGF-β1 in the fibrogenesis of the NIF mice, we treated the NIF mice with a blocking anti-TGF-β1 antibody (1D11). We found that treatment of the NIF mice with this antibody attenuated the fibrosis in the livers of the NIF mice (Fig. 5B,C). Therefore, we concluded that the development of liver fibrosis in NIF mice is dependent on TGF-β1 signaling.

### Fibrogenesis is associated with HSC activation and increased IL-33 expression

**Figure 6:**
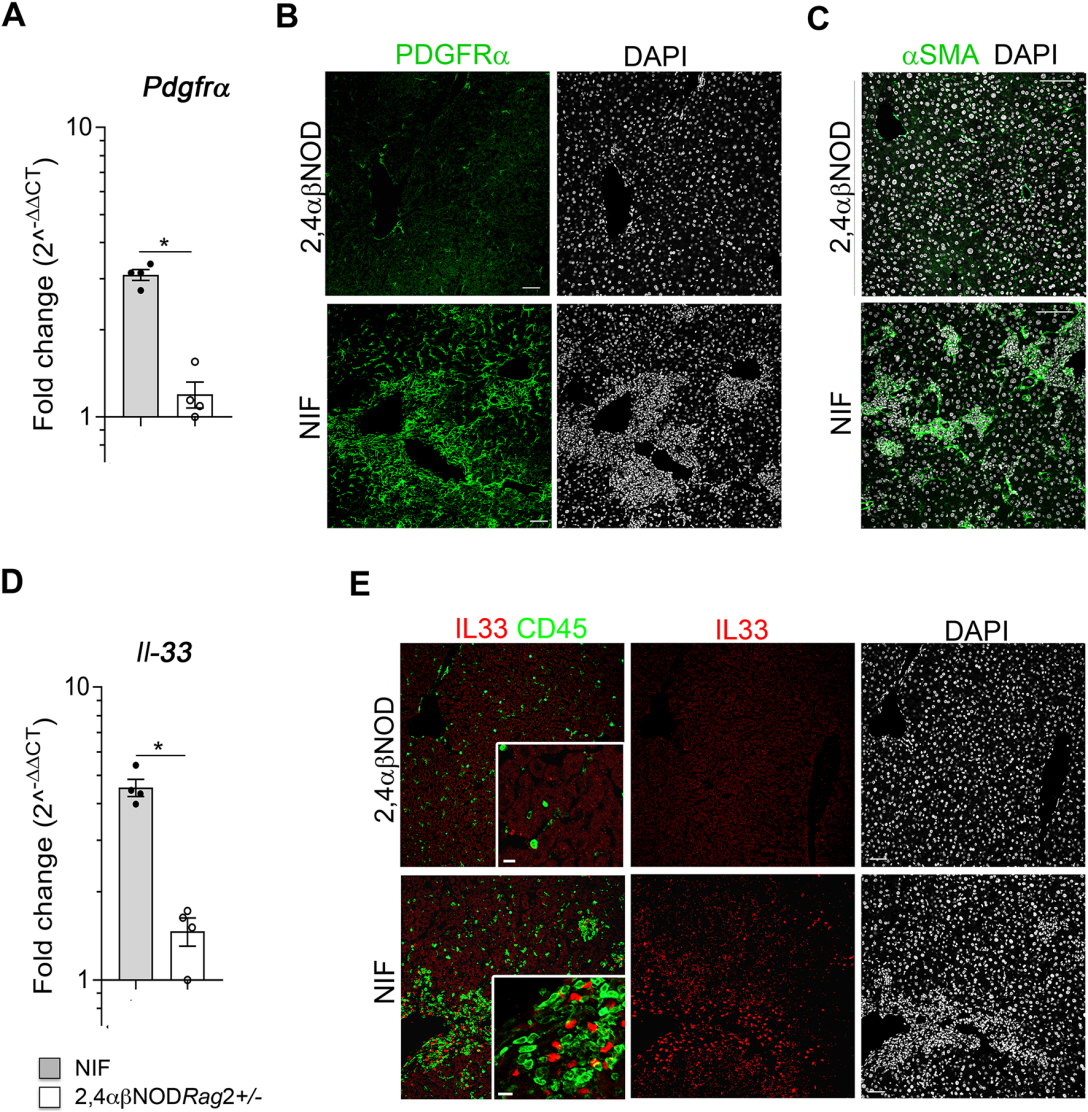
Liver inflammation is associated with the upregulation of IL-33 and the accumulation of activated hepatic stellate cells (HSCs) in the NIF mice. (**A**) RT-qPCR analysis of *Pdgfra* gene expression in the total liver lysates from the NIF mice (filled circle, shaded bars) (n = 4) and 2,4αβNOD.*Rag*2^+/−^ control mice (open circle, open bars) (n = 4). (**B**) Immunohistochemical staining of representative liver cryosections from 10-week-old NIF and 10-week-old 2,4αβNOD.*Rag*2^+/−^ control mice showing anti-PDGFRα (green) and DAPI (gray) staining. (**C**) Immunohistochemical staining of representative liver paraffin sections from 10-week-old NIF mice and 23-week-old 2,4αβNOD.*Rag*2^+/−^ control mice showing anti-αSMA (green) and DAPI (gray) staining. (**D**) RT-qPCR analysis of *Il-33* gene expression in the total liver lysates from NIF mice (filled circle, shaded bars) (n = 4) and 2,4αβNOD.*Rag*2^+/−^ control mice (open circle, open bars) (n = 4). (**E**) Immunohistochemical staining of the representative liver cryosections from 10-week-old NIF mice and 10-week-old 2,4αβNOD.*Rag*2^+/−^ control mice showing DAPI (gray) and anti-IL-33 (red) alone or merged with anti-CD45 (green) staining. Insets: enlarged area of inflammation illustrating that the proportion of IL-33^+^ cells increased with the age of the NIF mice and that the IL-33^+^ cells are CD45^-^. Scale bar: 50 µm for the large frames and 10 µm for the insets. *P < 0.05, **P < 0.01, and ***P < 0.001, Mann–Whitney U test after Bonferroni correction.

TGF-β1 is known to constitute an important factor in the activation of HSCs^38,39^. Its expression in NIF mice (Fig. 5A) together with the observed expression of CXCL1 and CCL2 (Fig. 3G–H), also known to activate HSCs, suggested that activation of the HSCs constituted a pathogenic factor in fibrogenesis in the NIF mouse livers. In support of this, we observed an increased expression of two markers known to be expressed by activated HSCs, platelet-derived growth factor receptor α (PDGFRα) and α-smooth muscle actin (αSMA). Thus, we observed the increased expression of the *Pdgfra* gene in the livers of the NIF mice (Fig. 6A) and an accumulation of PDGFRα^+^ HSCs in the NIF mouse livers (Fig. 6B). Similarly, we found an accumulation of αSMA^+^ cells in the fibrotic areas of the NIF mouse livers that increased with the age of the mice, up to 8 weeks of age, and correlated with fibrotic lesion development (Fig. 6C). We interpreted these findings as evidence of the presence of activated HSCs in the livers of the NIF mice.

IL-33 is generally expressed in the nucleus of endothelial and epithelial cells. It is released upon lysis of cells and acts as an alarmin, promoting a type 2 inflammatory reaction^40^. Recently, IL-33 has been increasingly associated with tuning of the inflammatory response towards repair and fibrogenesis. In the liver, IL-33 has been reported to be specifically expressed in injured hepatocytes and activated HSCs^13^ and to promote fibrosis development^41^. Therefore, we asked whether IL-33 plays a pathogenic role in liver fibrosis in NIF mice. As illustrated in Fig. 6, we found that, although the hepatocyte damage observed in the NIF mouse livers was modest, the IL-33 level was significantly increased in the livers of the 8-week-old NIF mice (Fig. 6D). Further, the immunohistochemical analysis revealed an accumulation of IL-33-expressing cells that correlated with the level of inflammation and reached a maximum in mice between 8 and 10 weeks of age (Fig. 6E). IL-33 was specifically detected in cells distributed in the areas of inflammation in the portal tracts but not preferentially in vascular endothelial cells. Moreover, no overlap in the expression of IL-33 and CD45 was detected, ruling out myeloid and lymphoid cells as the main sources of IL-33. Based on these observations, we concluded that IL-33 is mainly expressed by hepatocytes and/or HSCs in the livers of NIF mice.

### A switch in the NKT cell population to a predominantly type 2 cytokine production promotes the transition to type 2 inflammation in the NIF mice

**Figure 7:**
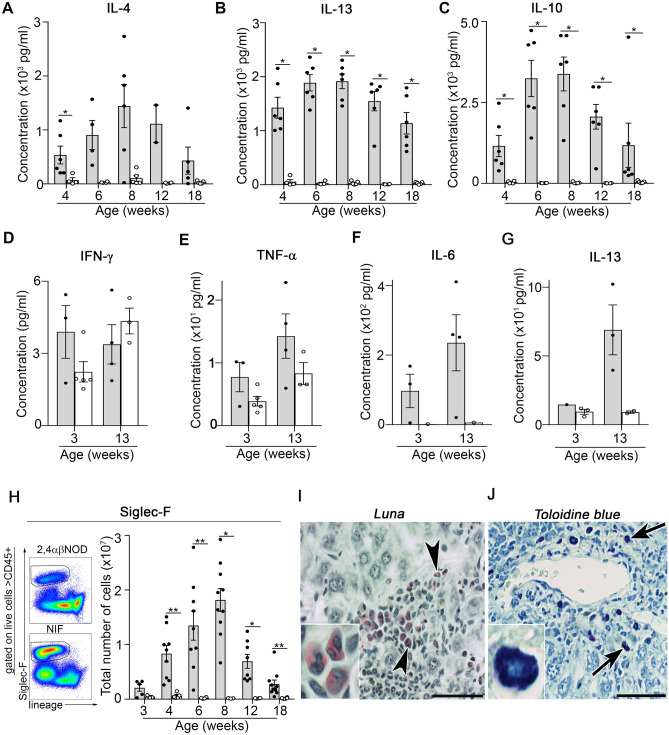
Transition to type 2 inflammation in the NIF mouse liver is associated with an altered cytokine profile in liver NKT cells. Levels of (**A**) IL-4, (**B**) IL-13 and (**C**) IL-10 in the supernatants of liver leukocytes stimulated for 24 h with anti-CD3 (4 µg/ml). NIF mice (filled circle, shaded bars) (n = 6) and 2,4αβNOD.*Rag*2^+/−^ control mice (open circle, open bars) (n = 3–5). Data are pooled from two independent experiments. Levels of (**D**) IFN-γ, (**E**) TNFα, (**F**) IL-6 and (**G**) IL-13 in the supernatants of sorted NKT cells isolated from 3 week old or 13 week old NIF and 2,4αβNOD.*Rag2*^*+/−*^ control mice and cultured for 24 h without stimulation. (**H**) Single cell suspensions from NIF and 2,4αβNOD.*Rag*2^+/−^ livers analyzed by flow cytometry. Representative dot plots (left) and the corresponding statistical analysis (right) are shown. Total number of Siglec-F^+^ cells gated from viable CD45^+^CD11b^+^Ly6G^-^ cells. *P < 0.05, **P < 0.01, and ***P < 0.001, Mann–Whitney U test after Bonferroni correction. Representative (**I**) Luna and (**J**) toluidine blue stained liver sections from 8-week-old NIF mice. Framed areas are highlighted. Scale bars are 200 μm.

Analysis of the cytokine profile suggested that the transgenic NKT cell population in the liver was modified in the NIF mice compared with that in the 2,4αβNOD.*Rag*2^+/−^ control mice. This was supported by the observed increase in the production of IL-4, IL-13 and IL-10 (Fig. 7A–C). To directly test whether a switch in the transgenic NKT cells occurred in the NIF mouse, we sorted NKT cells at different time points and analyzed their cytokine profile (Fig. 7D–G). We found that the NKT cells isolated from NIF mice displayed a mixed type1/type 2 cytokine profile at both 3 weeks and 13 weeks of age. However, we noted that while IFN-γ (Fig. 7D) was produced at a similar level in the two age groups, the production of IL-13 increased fourfold in the older age group (Fig. 7G). A less pronounced increase was also observed for TNFα (Fig. 7E) and IL-6 (Fig. 7F) in this age group.

In line with the transition to a predominantly type 2 cytokine profile, CD45^+^CD11b^+^SiglecF^+^ eosinophils were found to accumulate in the livers of NIF mice (Fig. 7H). In the histological analysis, we also noted marked eosinophilia (Fig. 7I) and accumulation of mast cells (Fig. 7J) in the livers of 8-week-old NIF mice.

Thus, the cytokine profile of the transgenic NKT cell population in young NIF mice was comparable to that of the 2,4αβNOD.*Rag*2^+/−^ control mice, indicating a progressive switch in the transgenic NKT cell population reflecting an anti-inflammatory, pro-repair/profibrotic cytokine profile in the adult NIF mice that was not observed in the 2,4αβNOD.*Rag*2^+/−^ control mice.

## Discussion

Relevant animal models are essential to extend our understanding of the mechanisms underlying sterile liver inflammation and fibrosis, including NAFLD and NASH, and to provide pertinent tools for preclinical drug testing. While several novel models of NAFLD/NASH that mainly reflect the metabolic phase of disease pathogenesis have recently been established^42^, models that accurately reproduce the inflammatory and fibrosis phase of the disease are still lacking^43^. Here, we show that overexpressing a transgenic population of NKT cells, in the absence of a functional adaptive immune system, induces chronic liver inflammation and promotes fibrogenesis in a novel mouse model, the NIF mice. We show how the accumulation of NKT cells in the livers of NIF mice precedes the recruitment of other inflammatory cells by approximately 2 weeks, suggesting that this NKT cell population mediates the initial events in liver pathogenesis. This notion is supported by the observation that NKT cells but not total spleen cells isolated from 2,4αβNOD.Rag2^+/−^ control mice could induce liver inflammation and fibrosis when adoptively transferred to naïve NOD.Rag2^−/−^ mice. We propose that the observed hepatocyte damage, albeit limited, together with the increased expression of pro-inflammatory cytokines such as TNFα and GM-CSF by the NKT cell population drives the initial type 1 inflammatory response in the livers of NIF mice. This process involves the activation of the NLRP3 inflammasome and processing of pro-caspase-1 and pro-IL-1β. The subsequent type 1 inflammatory response observed at the early stage of liver pathogenesis in the NIF mice largely overlaps with the initial stages of sterile liver inflammation associated with human liver diseases such as ALD and NAFLD/NASH.

The type 1 inflammatory response and the resulting tissue damage are assumed to be the driving forces behind the transition to a type 2 inflammatory response promoting tissue repair, when uncontrolled, this switch leads to fibrogenesis^9,44^. Alarmins constitute some of the earliest type 2 cytokines to be produced and contribute to this transition. Consistent with this finding, we observed a progressive transition into a type 2 inflammatory response in the NIF mice that included an accumulation of cells expressing the alarmin IL-33 in mice approximately 6 weeks old and older. IL-33 is known to activate ILC2s and drives a type 2 inflammatory response. The anti-inflammatory property of the type 2 cytokines is most likely reflected in the decreased magnitude of the pro-inflammatory response observed in the livers of NIF mice approximately 10–12 weeks old. It should be noted, however, that chronic inflammation, albeit at a lower level, characterized the livers of NIF mice at least 40 weeks of age. This outcome may be explained by the continuous output of newly formed NKT cells from the thymus. In addition, no signs of fibrosis remission were observed during the monitoring time, indicating that the fibrotic process is largely self-sustained. Consistent with this finding, activated αSMA^+^ HSCs were continuously observed in the liver.

Interestingly, we also observed a switch in the cytokine profile of the NKT cells in the NIF mice, leading to an increased production of type 2 cytokines particularly of IL-13, and to a distinct accumulation of eosinophils and other fibrosis-related cellular subpopulations in the liver^9^. NKT cells of both major types, iNKT cells and type 2 NKT cells, have been documented as producers of large quantities of different cytokines, depending on the local environment^27,45^. While iNKT cells have been mainly associated with a pro-inflammatory role and type 2 NKT cells mainly have a regulatory role, the plasticity of their cytokine production profiles suggests that each of these subsets can execute different functional roles^46^. The transgenic NKT cell population of the NIF mice, defined as type 2 NKT cells based on receptor specificity^34^, appears to drive the development of chronic type 1 liver inflammation and to support the shift of this into a type 2 inflammatory response, thus driving the development of progressive liver fibrosis in these mice. Prevailing models have proposed that the two major subsets of NKT cells, iNKT and type 2 NKT cells, play opposing roles in the control of inflammatory responses^20^. Hence, iNKT cells, representing the major population in the murine liver, have been associated with the production of pro-inflammatory cytokines and function mainly as promoters of type 1 inflammation. In contrast, type 2 NKT cells, representing the major NKT cell population in the human liver, preferentially produce type 2 cytokines and thus have been proposed to fulfill an anti-inflammatory role. Here, we observed that the transgenic NKT cell population, defined as type 2 NKT cells with respect to their TCR specificity, in the presence of a mature adaptive immune system acquires a predominantly pro-inflammatory cytokine profile. In contrast, in the immunodeficient background of the NIF mice, this cytokine profile shifts into a mixed type 1/type 2 cytokine profile. This mixed profile of the transgenic NKT cell population can be counteracted by the adoptive transfer of a naïve T cell population into the NIF mice^34^, which suggests that the development of hepatic inflammation and fibrosis is a reflection of the status of the T cell population, affecting the NKT cells and potentially other cell populations, such as different ILCs. The observed plasticity of the NKT cell population suggests that this cell population, depending on the local environment, can fulfill different roles in the coordination of the immune response.

As we reported previously^34^, the earliest detectable inflammation develops in the liver and skin of NIF mice approximately 3–4 weeks of age, while the first signs of inflammation in the kidneys and lungs occur much later. The early onset in the liver likely reflects the well-established preferential homing of NKT cells to the liver^46^. Based on these observations, we hypothesize that NKT cells may be key organizers of the balance between type 1 and type 2 inflammatory responses in the liver of NIF mice. In humans, increased numbers of NKT cells in the liver have been reported to be associated with sterile inflammation^47–49^ and to have both disease-promoting and disease-inhibiting effects on NAFLD and NASH development^21,30–32^. Several examples have been reported regarding how the local environment can reprogram the cytokine profile and function of both iNKT and type 2 NKT cells^46,50^. Moreover, a duality in the function of NKT cells as regulators of type 1/type 2 inflammatory responses has been previously reported in allergic airway disease^50^. Further analyses of the role of these subsets in the development of liver fibrosis are warranted and could open new avenues for identifying therapeutic targets.

Despite differences between the NIF mouse liver pathogenesis and that of human NASH, the similarities in the fibrogenic pathways and late-stage phenotype make the NIF mouse an interesting model for preclinical studies with potential translational opportunities. NASH has traditionally been believed to follow a “two-hit” course in which the first hit involves the development of steatosis, while the second hit involves the formation of an inflammatory and fibrotic response^51^. This view has been challenged, and a “multi-hit” model has been proposed suggesting that multiple factors act simultaneously to promote the development of NAFLD^52^. The type 1 inflammatory stage in NASH and in some of the animal models of the disease is driven by metabolic stress^42,53^. This component and its consequences in terms of liver steatosis and shifting of the system towards the production of IL-1β, TNFα, IL-6 and IL-12 are missing in NIF mice. However, while the etiology and earliest stages of disease development in NIF mice are distinct from NASH in humans, we found that the transgenic NKT cell population in the liver promotes a similar process in NIF mice. From this point in the pathogenesis, disease progression through the expression of TGF-β1, together with IL-4 and IL-13, drives the fibrogenic pathway described for human NASH. Despite the absence of liver steatosis and associated phenomena, such as hepatocyte ballooning, the extensive inflammation and fibrosis that develop in the livers of the NIF mice were accompanied by histopathological evidence of hepatocyte damage with focal necrosis and bile duct hyperplasia. We noted that this development was associated with a moderate but significant increase in serum bile acids but an unexpected lack of changes in the liver enzymes AST/ALT. Thus, while the lack of metabolically related etiology and early pathology precludes the analysis of therapies targeting the earliest events in the disease, the NIF mice constitute an improved model and useful tool for testing specific targets represented in the type 2 inflammatory response and fibrogenesis.

## Methods

### Mice

All animals were housed under specific pathogen**–**free conditions at the Biomedical Center animal facility in Lund, Sweden. To generate 2,4αβNOD.*Rag2*^−/−^ (NIF) mice, 2,4αβNOD.*Rag2*^−/−^ male mice were bred with female 2,4αβNOD.*Rag2*^+/−^ generating both NIF and 2,4αβNOD.*Rag2*^+/−^ control mice. The 2,4αβNOD.Rag2^−/−^.CD2-GFP (NIF.CD2-GFP) mouse was generated by crossing the NIF mouse and the NOD.CD2-GFP mouse^35^. NOD.CD2-GFP mice were a gift from Dr Anne Cooke (Department of Pathology, University of Cambridge, United Kingdom).

### Antibody treatment regimen

6 weeks old male NIF mice were treated with 5 mg/kg of 1D11 (Nordic BioSite, Sweden) or PBS 3 times/week for 4 weeks. Each reagent was administered by sterile intraperitoneal injection over a 4-week period.

### Histology

Mice were sacrificed by cervical dislocation and livers were perfused with PBS via the inferior vena cava. Preparation of paraffin-embedded liver sections and staining with hematoxylin and eosin (H&E) or picrosirius red were performed as described previously^34^. The sections were treated for antigen retrieval by high-temperature heating in Tris–EDTA buffer and stained using primary antibody against αSMA and secondary anti-rabbit Alexa 488 antibodies.

Immunohistochemical staining of frozen tissue was performed as previously described^54^. Eight-micrometer frozen sections were stained overnight with primary anti-IL-33, anti-PDGFRα or anti-CD45 antibodies at 4 °C followed by incubation with appropriate secondary antibodies conjugated with Alexa 488/594 fluorophores from Life Technologies (Thermo Fisher Scientific, Darmstadt, Germany) for 1 h at room temperature (1:500). Digital images of the sections mounted with fluorescence mounting medium (Dako, Glostrup, Denmark) were acquired with a Zeiss LSM 800 Airyscan confocal laser-scanning microscope.

### Pathological staging

An ECVP board certified pathologist used a light Leitz Diaplan microscope to assess H&E and PSR-stained liver sections according to a classical 5-point semiquantitative scoring scale of the lesions. In addition, a six-point Ishak scoring scale was used to evaluate the PSR-stained liver sections.

### Quantitative assessment of fibrosis

Digital images of the PSR-stained sections were acquired with a Panoramic 250 Flash II scanner (3DHistech, Hungary). For the quantitative assessment of these images, a semiautomated algorithm was used to extract detailed morphometric data on the red-stained collagen using the automatic thresholding function in ImageJ (run as a macro).

### Hydroxyproline measurement

As previously described^33^ the hydroxyproline content of the liver was determined using a hydroxyproline colorimetric assay kit (BioVision, Milpitas, CA, USA) according to the manufacturer’s instructions. Briefly, 100 μl of wet tissue (10 mg) was hydrolyzed in 100 μl of 12 N HCl for 3 h at 120 °C. The aliquots were transferred to a 96-well plate, dried for 1.5 h at 60 °C and incubated with chloramine T and DMAB reagents for 90 min at 60 °C. Finally, the absorbance at 560 nm was measured.

### Cytokine analysis

Cytokine analyses were performed as previously described^33^. Single cell suspensions of leukocytes isolated from the liver (10^5^ per well) or sorted NKT cells from the liver (2.5 × 10^5^ per well) were cultured in complete RPMI 1640 medium supplemented with 10% FCS, 100 U/ml penicillin/streptomycin, 2.5% sodium bicarbonate (7.5% solution), 1 mM sodium pyruvate and 69 µM 1-thioglycerol unstimulated or activated with an anti-CD3 antibody (4 µg/ml, clone 154-2C11, BD Biosciences). The supernatants were collected after 24 h in culture and stored at −80 °C before being analyzed for cytokines using the mouse Th1/Th2/Th9/Th17/Th22/Treg Cytokine 17-plex Mouse ProcartaPlex Panel (Invitrogen) according to the manufacturer’s instructions.

Plasma was collected using EDTA-coated microcentrifuge tubes and centrifuged for 15 min at 1000 × g. The cleared plasma was collected and stored at −80 °C before being analyzed for cytokines. For cytokine measurements in plasma or culture supernatants of sorted NKT cells, the MSD U-PLEX platform (Meso Scale Diagnostics, Rockville, MD, USA) was used according to the manufacturer’s instructions.

### Gene expression analysis

As previously described^33^, RNA was extracted from liver biopsy samples by the homogenization of tissue in QIAzol lysis reagent (Qiagen, Hilden, Germany), followed by purification on RNeasy Mini columns (Qiagen) according to the manufacturer’s protocol. Total RNA (0.5 μg) was reverse transcribed using a RT^2^ HT First Strand kit (Qiagen). Genomic elimination was carried out in a C1000 Thermal Cycler (Bio-Rad, Hercules, CA) at 42 °C for 5 min. Next, cDNA synthesis was performed in the C1000 Thermal Cycler at 42 °C for 15 min, and the reaction was terminated at 95 °C, which required 5 min for completion. The cDNA was used for quantitative PCR analysis using RT^2^ SYBR Green Master Mix (Qiagen) at 95 °C for 10 min, followed by 40 cycles at 95 °C for 15 s and 60 °C for 1 min in a ViiA 7 thermal cycler (Applied Biosystems). The analyses were performed using primers for mouse *Nfkb, Nlrp3*, *Casp1, Il1b, Cxcl1, Ccl2, Tgfb1, Pdgfra,* and *Il33. Gapdh* was used as a housekeeping gene (see 1 Table S1 for details). Fold changes in mRNA expression of the genes were calculated by using the ΔΔCt method. The relative normalized expression of each gene for each sample was calculated using the formula 2^-ΔΔCt^, with the expression data from 8 week old 2,4αβNOD.*Rag2*^*+/−*^ mice used as the controls.

### Flow cytometry

Flow cytometric analyses were performed as previously described^34^. Liver leukocytes were obtained by incubating minced pieces of liver in 1.0 mg/ml collagenase II solution (Sigma) for 20 min at 37 °C, after which the tissue was pushed through a 70 µm mesh, and the leukocytes were separated on a 50/30% Percoll gradient (GE Healthcare) by centrifugation. The isolated cells were stained in FACS buffer (3% FCS in PBS) as follows: prior to surface staining, the cells were incubated with 2.4G2 (anti-CD16/CD32) Ab (BD Biosciences) to prevent nonspecific binding. The cells were then incubated with fluorochrome-conjugated anti-mouse antibodies specific for the cell surface markers listed in Table S1. The stained cells were then analyzed using a BC CytoFLEX flow cytometer and FlowJo software (TreeStar, Ashland, OR).

### Adoptive transfer experiments

Live CD2-GFP^high^Vα3.2^+^Vβ9^+^NKT cells were sorted from spleens of 12 week old male or female 2,4αβNOD.CD2-GFP donor mice using a BD FACSAria Fusion (nozzle size 100 μm) (Table S1). Purity of the sorted cells was > 97.5%. 1.3 × 10^5^ sorted tgNKT cells or 1 × 10y ^7^ total spleen cells were then adoptively transferred to 4 week old NOD.*Rag2*^*−/−*^ recipient mice. For endpoint analysis, mice were sacrificed 10 weeks post adoptive transfer and livers were analyzed by Flow cytometry as described above using an alternative staining panel (Table S1) and Immunofluorescence analysis.

### Immunoblot analysis

To separate the intracellular proteins from extracellular proteins in the mouse liver, extracted livers were transferred to a 70 µm strainer and subjected to complete protease inhibitor cocktail (Roche) in 2.5 ml of PBS per 1 g tissue. The cells in each 1 ml suspension were centrifuged at 1000 × g and 4 °C for 5 min to separate the extracellular proteins (in the supernatant). The supernatants were transferred to new prechilled 1.5 ml reaction tubes, mixed at a ratio of 1:20 with 2 × reducing Laemmli buffer and then heated at 95 °C for 10 min.

The cell pellets were resuspended in 5 ml of 1 × red blood cell (RBC) lysis buffer (Santa Cruz) and incubated for 5 min at room temperature. After RBC lysis, the cells in 150 µl of cell suspension were pelleted at 1000 × g and 4 °C for 5 min. The cell pellets were resuspended in 200 µl of lysis buffer (20 mM Tris–HCl, pH 7.4; 150 mM NaCl; 1 mM EDTA; 1% NP-40; 0.5% deoxycholate; and 0.2% SDS containing phosphatase inhibitor (Biotool) and cOmplete protease inhibitor cocktail (Roche) and incubated on ice for 10 min, followed by centrifugation at 20,000 × g and 4 °C for 10 min. The cleared lysates were mixed in a ratio of 3:1 with 6 × reducing Laemmli buffer and heated at 95 °C for 10 min.

The proteins in the supernatant and lysate (cell pellet) samples were separated on NuPAGE 10% Bis–Tris gels (Invitrogen; Thermo Fisher Scientific) and immunoblotted onto nitrocellulose membranes (Amersham). The membranes were blocked for 1 h in 1 × Roti Block (Roth) and subsequently incubated with different primary antibodies in TBS-Tween/5% BSA overnight.

The following primary antibodies were used: goat anti-IL-1β from R&D Systems (#AF-401-MA; 1:2,500 dilution); NLRP3 rabbit monoclonal antibody (D4D8T) from Cell Signaling Technology (#15101; 1:1,000 dilution); and mouse anti-Caspase-1 (p20) monoclonal antibody (Casper-1) obtained from Adipogen (#AG-20B-0042; 1:1,000 dilution).

After incubation with HRP-labeled secondary antibodies (either in 1 × Roti Block or TBS-Tween/2.5% BSA), the proteins were detected using ECL substrates and X-ray film from GE Healthcare Amersham. The following secondary antibodies were used: anti-HRP-labeled secondary anti-rabbit antibody from Cell Signaling Technology (#7074; 1:10,000 dilution); anti-HRP-labeled secondary anti-goat antibody from Santa Cruz Biotechnology (#sc-2020; used 1:10,000 dilution); and mouse TrueBlot ULTRA anti-mouse Ig HRP (eB144) from Rockland (#18-8817-33; 1:1,000 dilution).

### Serum markers

Serum was collected from clotted whole blood by centrifugation for 10 min at 1500 × g. The cleared supernatant was collected and analyzed at The University Animal Hospital, SLU, Uppsala for AST, ALT, triglycerides and bile acid using a fully automated Architect c4000 (Abbott Laboratories, Abbott Park, IL, US).

### Statistics

The results are presented as the means and standard error of the mean (SEM). Differences between two groups were considered significant when *P* < 0.05, as assessed using the Mann–Whitney U test and Bonferroni corrections by GraphPad Prism 7 software (San Diego, CA).

### Study approval

Animal experiments were performed in strict accordance with the recommendations for the use of laboratory animals from the Swedish Board of Agriculture and were approved by the ethics committee of animal experiments of Malmö/Lund (permit no. M143-11 and no. M52-16). The mice were sacrificed by cervical dislocation. All efforts were made to minimize animal suffering.

## Supplementary information

Supplementary Information.

## Data Availability

All data generated or analyzed during this study are included in this published article (and its Supplementary Information files).
