# Supplementary material for: NKT cells promote both type 1 and type 2 inflammatory responses in a mouse model of liver fibrosis
Source: Sci Rep. 2020 Dec 11;10:21778. doi: 10.1038/s41598-020-78688-2 (PMC7732838; doi:10.1038/s41598-020-78688-2)
Supplement: Supplementary file 1 — Supplementary Information. [file 41598_2020_78688_MOESM1_ESM.pdf]

Supporting information:

NKT cells promote both type 1 and type 2 inflammatory responses in a mouse model for liver fibrosis.

Julia Nilsson, Maria Hörnberg, Anja Schmidt-Christensen, Kajsa Linde, Maria Nilsson, Marine Carlus, Saskia F.Erttmann, Sofia Mayans, and Dan Holmberg

Fig. S1. Related to Figure 1. Male and female liver weight/body weight ratio

Fig. S2. Related to Figure 1. Bile duct hyperplasia

Fig. S3. Related to Figure 1**.** Serum levels of liver enzymes

Fig. S4. Related to Figures 2, 3 and 7. Full gating strategy.

Fig. S5 Related to Figure 2. Composition of subsets of $CD45^{+}$ cells in liver and spleen

Fig. S6. Related to Figures 2. Characterization of the NIF.CD2-GFP mouse.

Fig. S7. Related to Figures 2. Adoptive transfer of tgNKT II cells promotes inflammation in livers of naïve $NOD.Rag2^{-/-}$ recipients.

Fig. S8. Related to Figure 3. Uncropped/unedited images for all blots and gel

Fig. S9. Related to Figures 4 and 5. Quantification of PSR staining.

Supplementary Table 1. List of reagents and resources used in this study

Fig S1. Related to Figure 1.

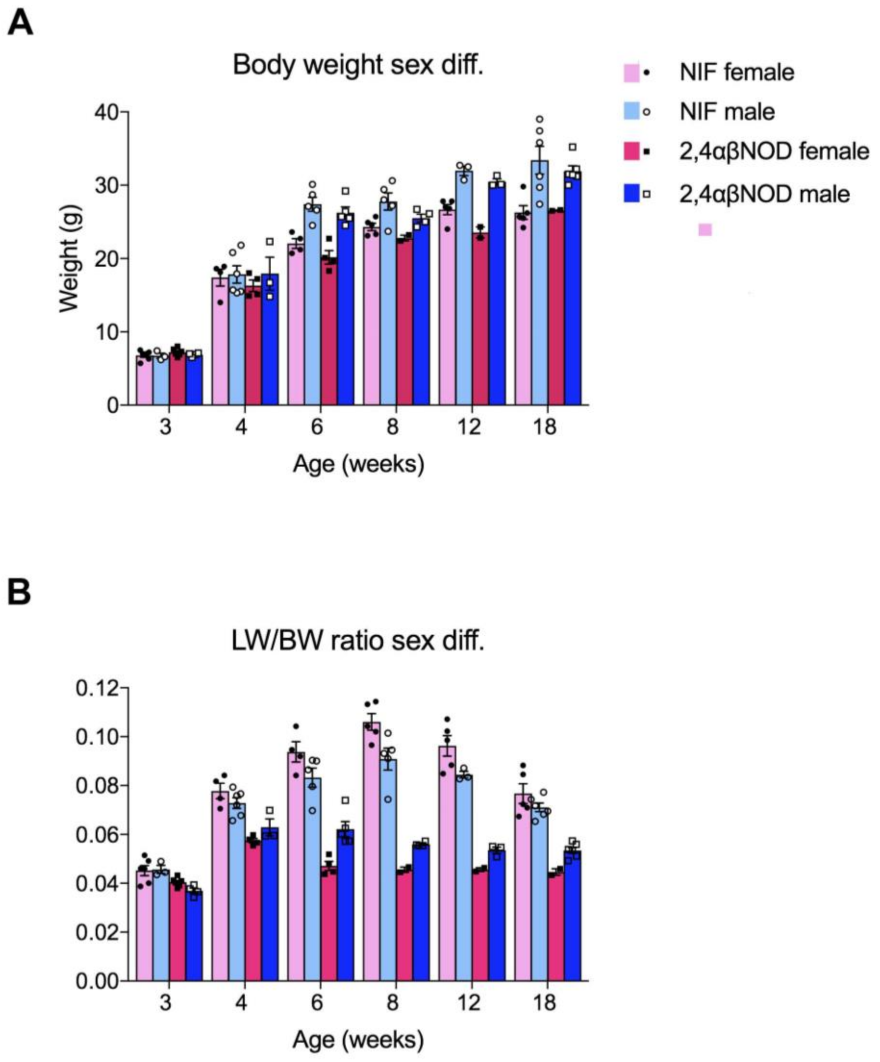

Fig S1. Male and female liver weight/body weight ratio

Male NIF mice gained more weight than females but no gender difference were observed in liver to body weight ratio in the NIF mouse. Female NIF mice (pink bars and ●) (n = 4-6), female 2,4αβNOD.*Rag*2$^{+/-}$ control mice (fuchsia bars and ▪)(n = 2-6), male NIF mice (light blue bars and ○) (n = 3-6) and male 2,4αβNOD.*Rag*2$^{+/-}$ control mice (dark blue bars and) (n = 3-6).

Fig S2. Related to Figure 1.

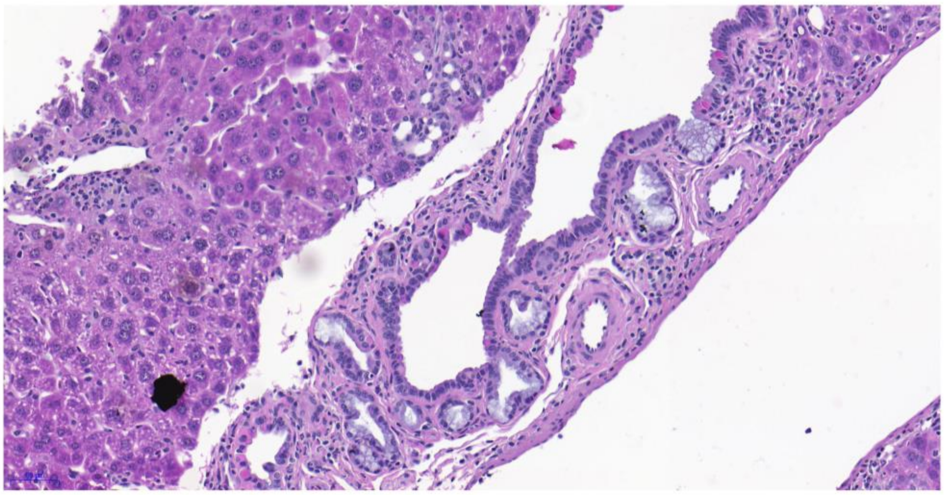

Fig S2. Bile duct hyperplasia
Eosin & hematoxylin staining of liver section from 12-weeks-old NIF mouse illustrating bile duct hyperplasia.

Fig S3. Related to Figure 1.

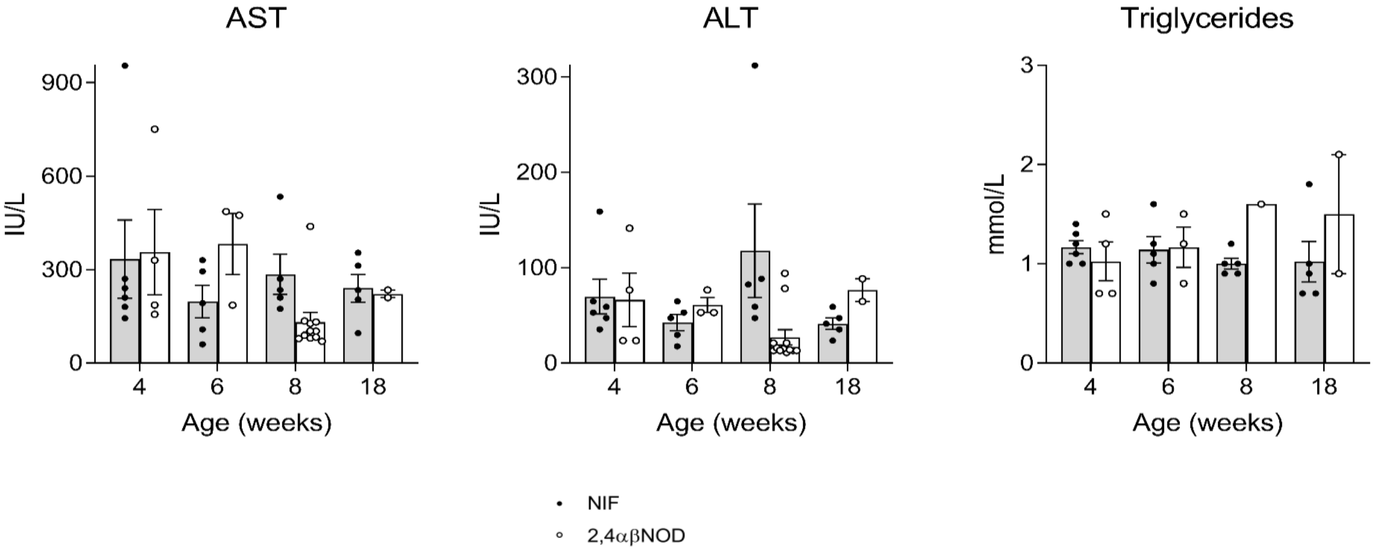

Fig S3. Serum levels of liver enzymes

Serum levels of liver markers, aspartate aminotransferase (AST), alanine aminotransferase (ALT), and triglycerides in NIF (n=4-6) and 2,4αβNOD control mice (n=2-10). Mice were bled at the indicated ages and serum was collected and sent to SLU, Uppsala for measuring AST, ALT, and TG using a fully automated Architect c4000 (Abbott Laboratories, Abbott Park, IL, US).

Fig S4. Related to Figures 2, 3 and 7.

A

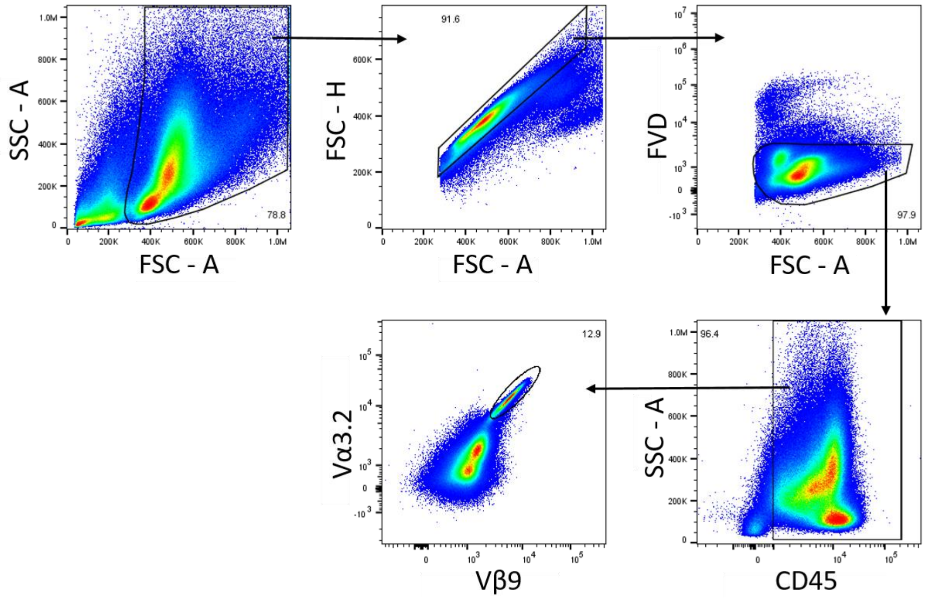

B

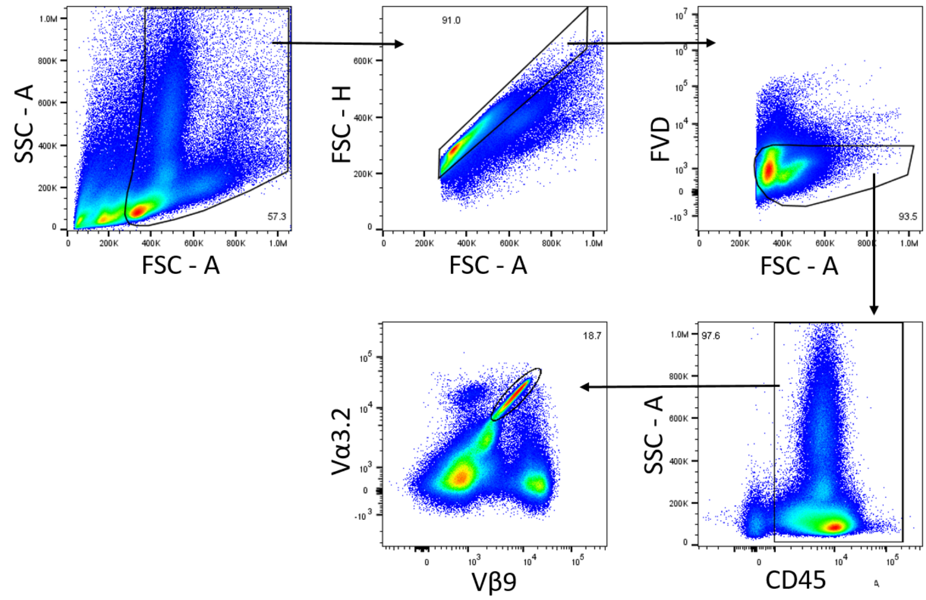

C

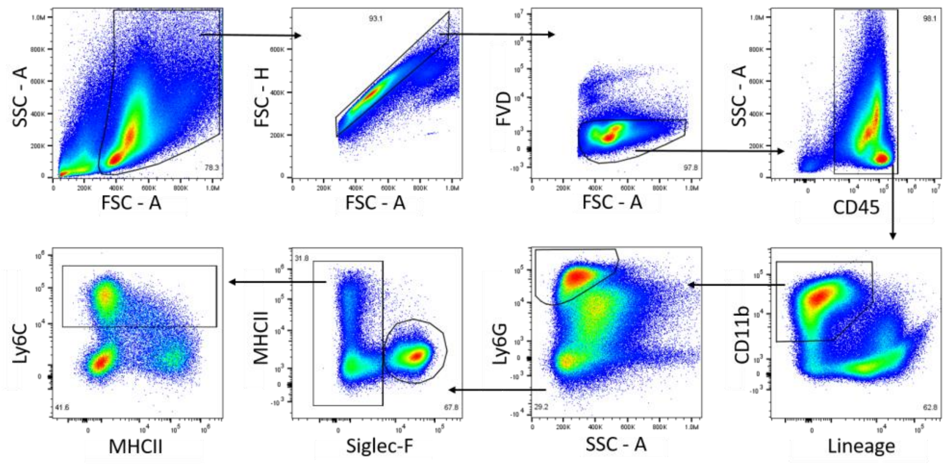

D

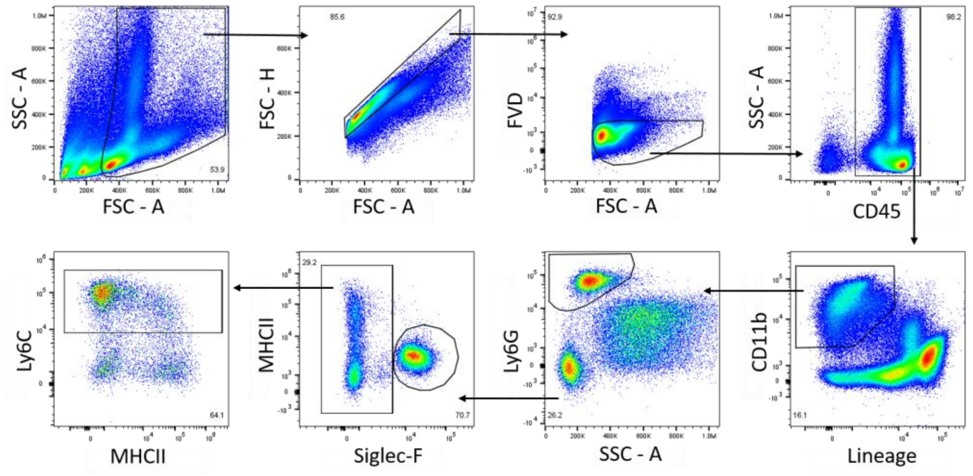

Fig S4. Full gating strategy

Gating strategy for liver transgenic NKT cells of (A, C) NIF and (B, D) 2,4αβNOD.*Rag2*$^{+/-}$ control mice. Doublets were excluded using strict SSC-A/SSC-H and FSC-A/FSC-H gates. Live/dead gating was performed after FSC/ fixable viability dye. Leukocytes were defined by the expression of CD45. (A, B) The transgenic NKT cells were defined by its T cell receptor (Vα3.2/Vβ9) and (C, D) eosinophilic/neutrophilic granulocytes by the CD11b expression along with SiglecF (for eosinophils) and Ly6G (for neutrophils).Linage includes CD3, CD19, TER119, CD49b, B220, CD8a, CD4

Fig. S5 Related to Figures 2

A. Cellular composition in liver

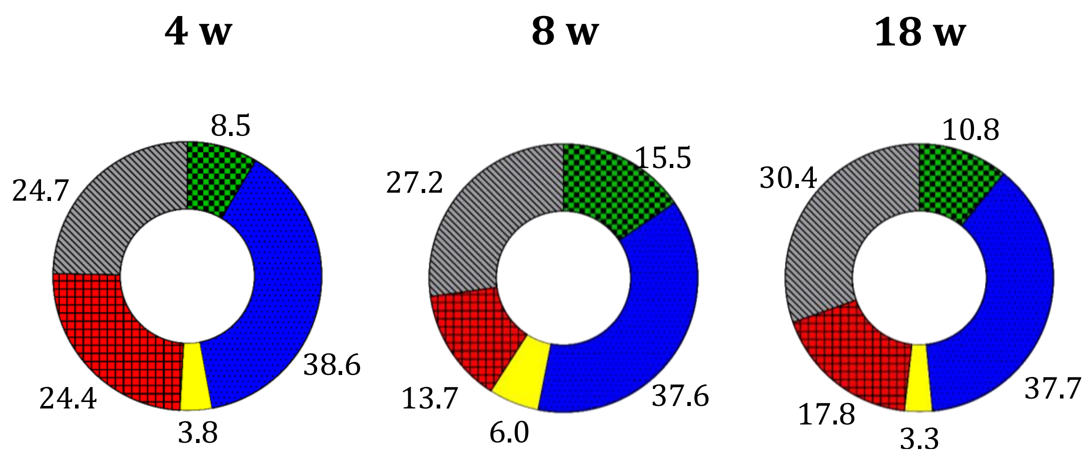

B. Cellular composition in spleen

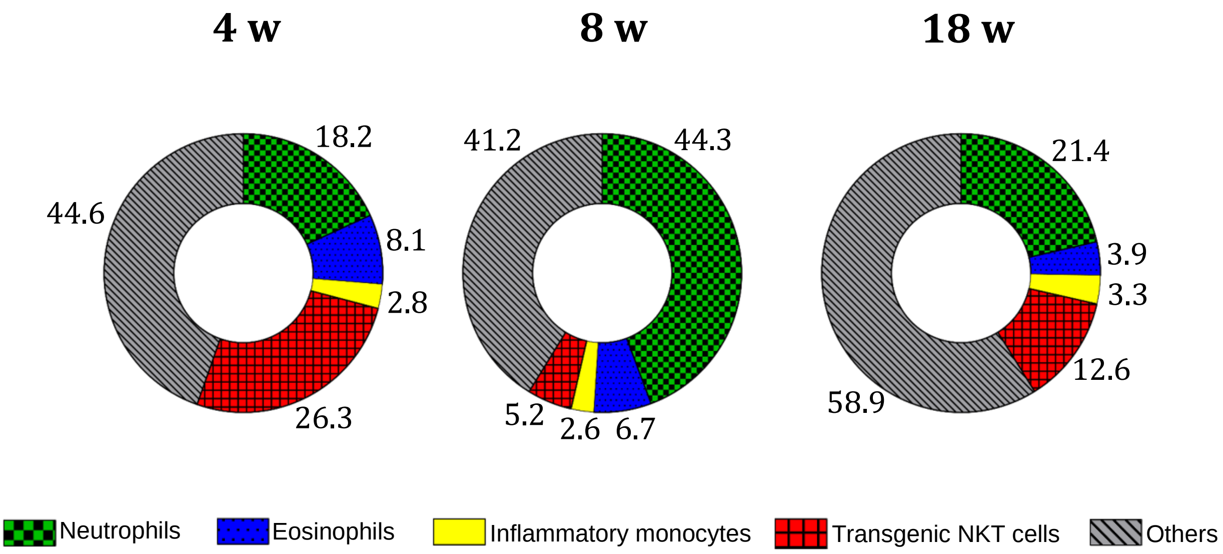

Fig S5. Composition of subsets of CD45⁺ cells in liver and spleen
Pie charts showing the frequency of Neutrophils (Ly6G⁺), Eosinophils (Siglec-F⁺), Inflammatory monocytes (Ly6C⁺) and transgenic NKT cells (Vα3.2⁺, Vβ9⁺) among CD45⁺ cells isolated from NIF (A) liver (n = 9-10) and (B) spleen (n = 8-11). Data pooled from 17 independent experiments.

Fig. S6 Related to Figure 2.

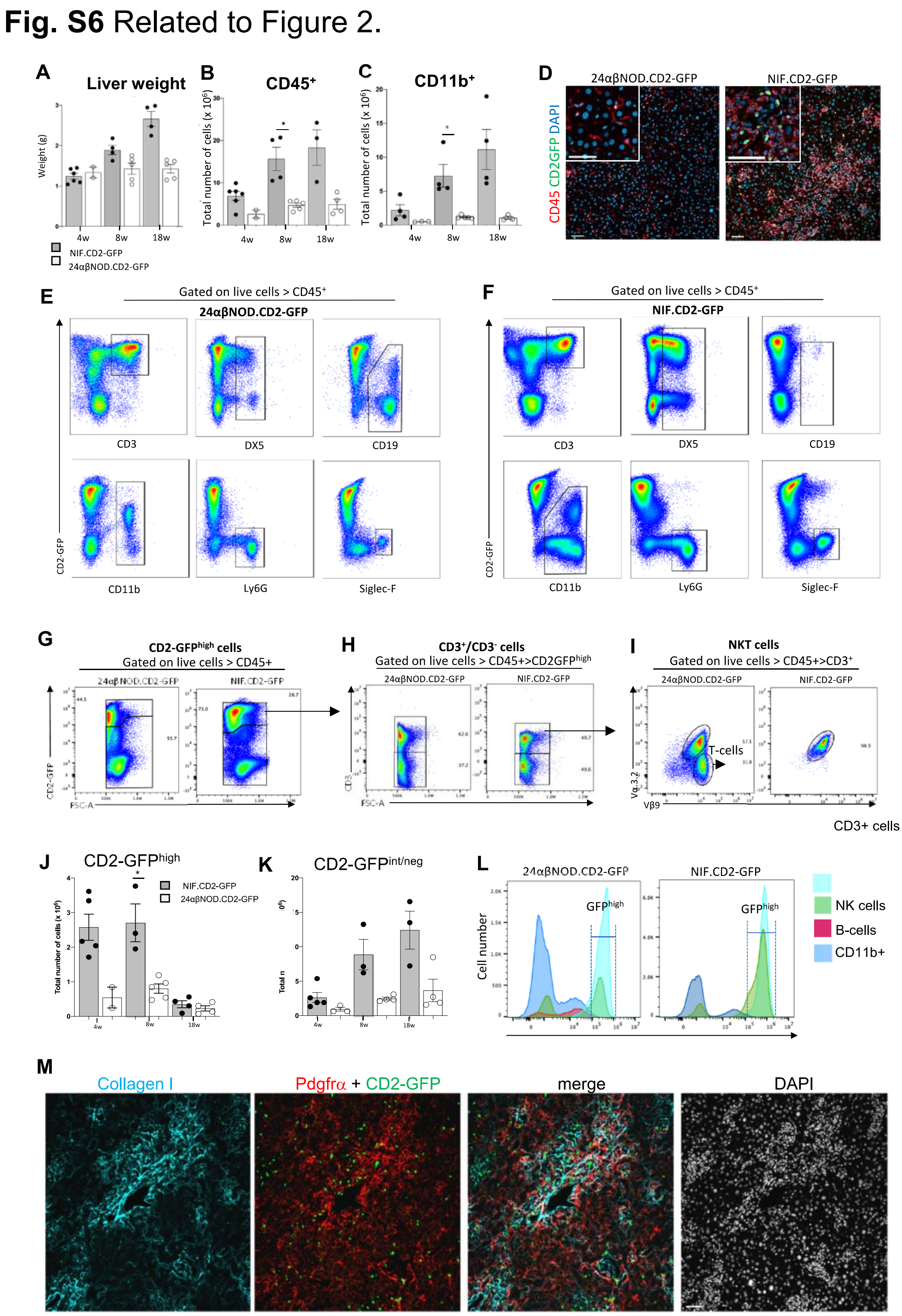

Fig.S6: Characterization of the NIF.CD2-GFP mouse.

A-C: Kinetics of liver inflammation in NIF.CD2-GFP mice. **A:** Liver weight of 4, 8 and 18-week old NIF.CD2-GFP (●)(n=4-6) and 24αβNOD.Rag2$^{+/-}$.CD2-GFP control mice (○) (n=2-5). **B**: Flow cytometry analysis of single cell suspensions from NIF.CD2-GFP and 24αβNOD. Rag2$^{+/-}$.CD2-GFP livers showing total number of CD45^{+} cells (**B**) and CD11b^{+} cells (**C**) per analyzed liver. **D**: Cryo-sections of liver tissue from 8-week old female 24abNOD.Rag2$^{-/-}$.CD2-GFP or NIF.CD2-GFP mice stained for CD45 (red) and DAPI (blue). Endogenous CD2-GFP expression is shown in green. Scale bars are 50 µm in the overview images (right) and 10 µm in the highlighted images (left). **E-I**: Characterization of 8-week old 24αβNOD.Rag2$^{+/-}$.CD2-GFP control (**E**) or NIF.CD2-GFP mice (**F**) for the endogenous expression of CD2-GFP in CD3^{+} T/NKT cells, DX5^{+} NK cells, CD19^{+} B cells, CD11b^{+} myeloid cells, Ly6G^{+} cells or SiglecF^{+} eosinophils. Within the live CD45^{+}CD2-GFP^{high} population (indicated in **G**), CD3 expression (**H**) was used to further discriminate CD2-GFP^{high}CD3^{-} NK cells and transgenic Vα.3.2^{+}Vβ9^{+}CD3^{+} NKT II cells (**I**). **J-L**: Kinetics of liver leukocytes with high (CD2-GFP^{high}) compared to intermediate/negative (CD2-GFP$^{int/neg}$) expression level. Quantification of CD2-GFP^{high} (**J**) and CD2-GFP$^{int/neg}$ (**K**) cells (indicated in **L**) in NIF.CD2-GFP (shaded bars and □, n=3-5) and 24αβNOD.CD2-GFP (open bars and (○), n=2-5) livers measured by Flow cytometry. Data are pooled from 8 independent experiments; *P<0,05, **P<0,01 and ***P<0,001. Mann-Whitney nonparametric, unpaired two-tailed rank test was used as statistic test and results are presented as mean ± SEM. **L**: Histogram showing GFP intensity in CD2-GFP^{+} leukocyte subsets in livers of 8 week old 24αβNOD.Rag2$^{+/-}$.CD2-GFP or NIF.CD2-GFP mice; CD3^{+} T/NKTcells (light blue), NK cells (green), CD19^{+} B-cells (red) and CD11b^{+} cells (dark blue). **M:** Fibrosis development in NIF.CD2-GFP mice. Representative immunofluorescence images illustrating the liver portal area in 8-week old NIF.CD2-GFP mice stained for collagen I (cyan), PDGFRα (red) and DAPI (grey). Endogenous CD2-GFP is shown in green. Images of collagen I and DAPI alone or merged with PDGFRα and CD2-GFP. Scale bars in D,M: 50 µm.

Fig. S7. Related to Figure 2.

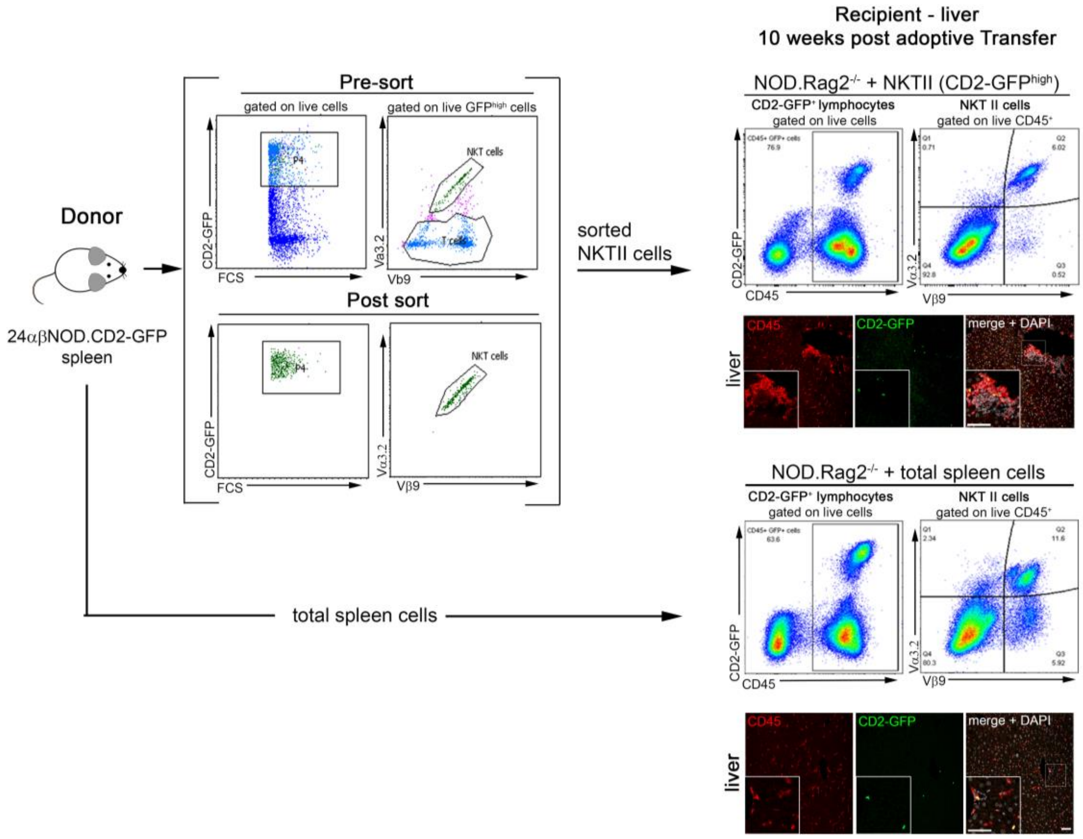

Fig. S7: Adoptive transfer of tgNKT II cells promotes inflammation in livers of naïve NOD.Rag2$^{-/-}$ recipients.

Sorted tgNKT II cells or total spleen cells from female or male 12-week old 24abNOD.Rag2$^{+/-}$.CD2-GFP healthy donor mice were adoptively transferred to naïve 4-week old NOD.Rag2$^{-/-}$ recipient mice and recipient livers were analyzed 10 weeks post adoptive transfer. (**A**) FACS plots showing the gating strategy (live CD2-GFP^{high} > Vα3.2^{+}/Vβ9^{+}) for the sorting of tgNKT II cells (pre-sort) and sorted CD2-GFP^{high} Vα3.2^{+}Vβ9^{+}NKT II cells (post-sort) used for adoptive transfer to NOD.Rag2$^{-/-}$ recipient mice. (**B-E**) Livers of NOD.Rag2$^{-/-}$ recipient mice injected with either 1.3x10^{5} purified splenic tgNKT II cells (**B,C**) (n=5 mice, incl. 2 females and 3 males) or 1 x10^{7} total spleen cells (**D,E**) (n=3 mice, incl. 2 males and 1 female) were analyzed by Flow cytometry or Immunoflourescence. (**B,D**): Plots with frequencies showing reconstituted live CD2-GFP^{+} lymphocytes from recipient livers. In recipients with transferred sorted tgNKT cells this CD2-GFP population consists predominantly of live CD45^{+}Vα3.2^{+}Vβ9^{+}NKT II cells (**B**), whereas control recipient livers contain additionally other CD2-GFP^{+} T cell populations (live CD45^{+}Vα3.2^{-}Vβ9^{+}) (**D**). **C,E**: Liver cryo-sections from NOD.Rag2$^{-/-}$ recipient mice 10 weeks post adoptive transfer with purified tgNKT II cells (**C**) or total spleen cells (**E**) stained with anti-CD45 (red) and DAPI (grey). CD2-GFP positive cells are indicated in green. Scale bar: 50µm.

Fig S8 Uncropped/unedited images for all blots and gels included in Figure 3F.

Liver cell pellet samples:
Primary AB: anti-mouse Caspase-1 (Casper: 1:1000)
Secondary AB: Mouse TrueBlot ULTRA: anti-mouse Ig HRP (1:1000)

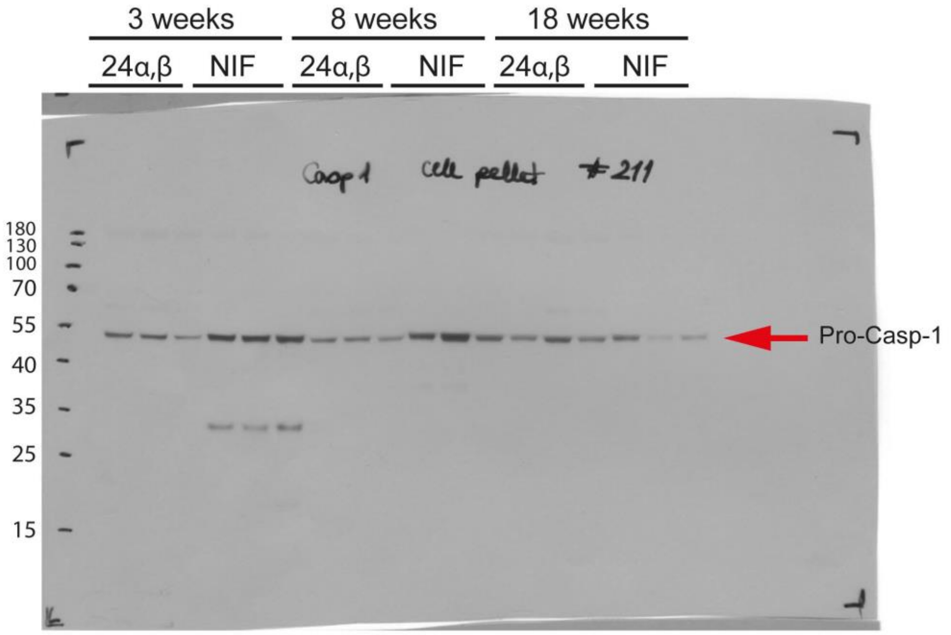

Liver cell pellet samples:
Primary AB: anti-mouse IL-1β (1:2500)
Secondary AB: HRP-labelled anti-goat antibody (1:10000)

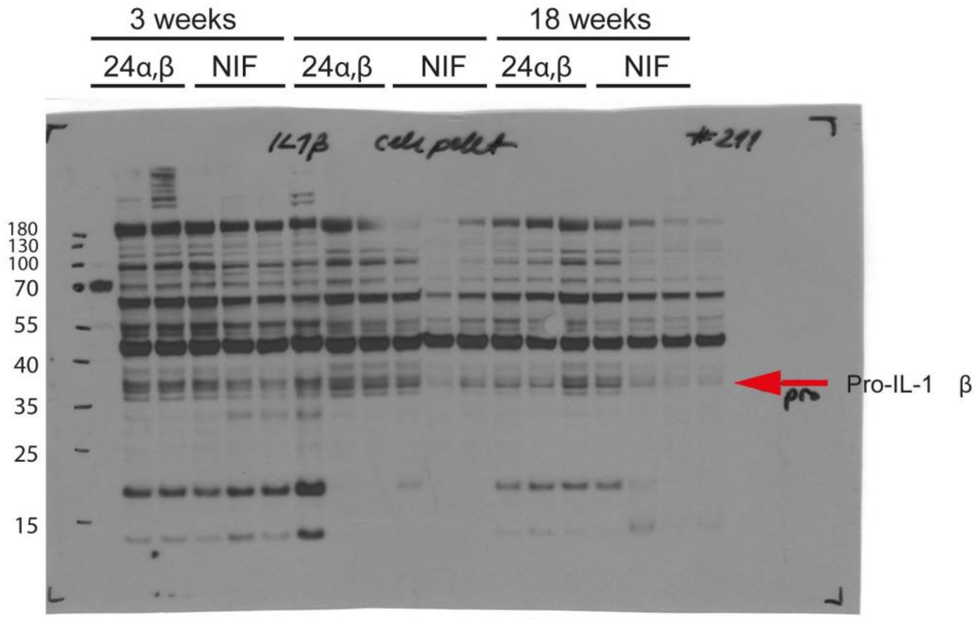

Liver cell pellet samples (stripped menbrane from IL-1β)
Primary AB: anti-NLRP3 (1:1000)
Secondary AB: HRP-labelled anti-rabbit antibody (1:10000)

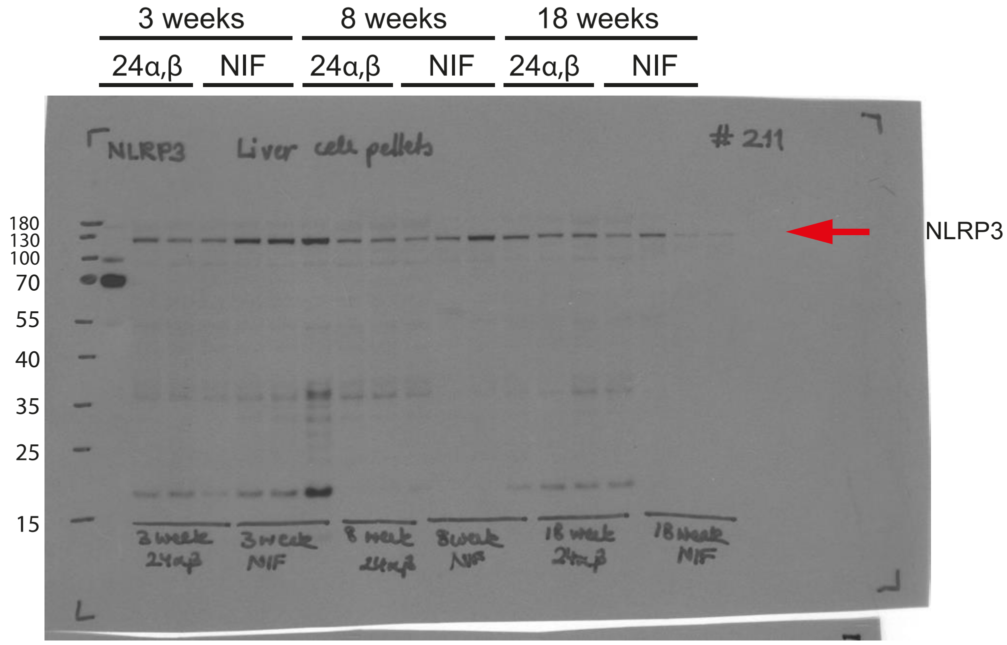

Liver cell pellet samples:
Unspecific band as loading control
Primary AB: anti-mouse IL-1β (1:2500)
Secondary AB: HRP-labelled anti-rabbit antibody (1:10000)

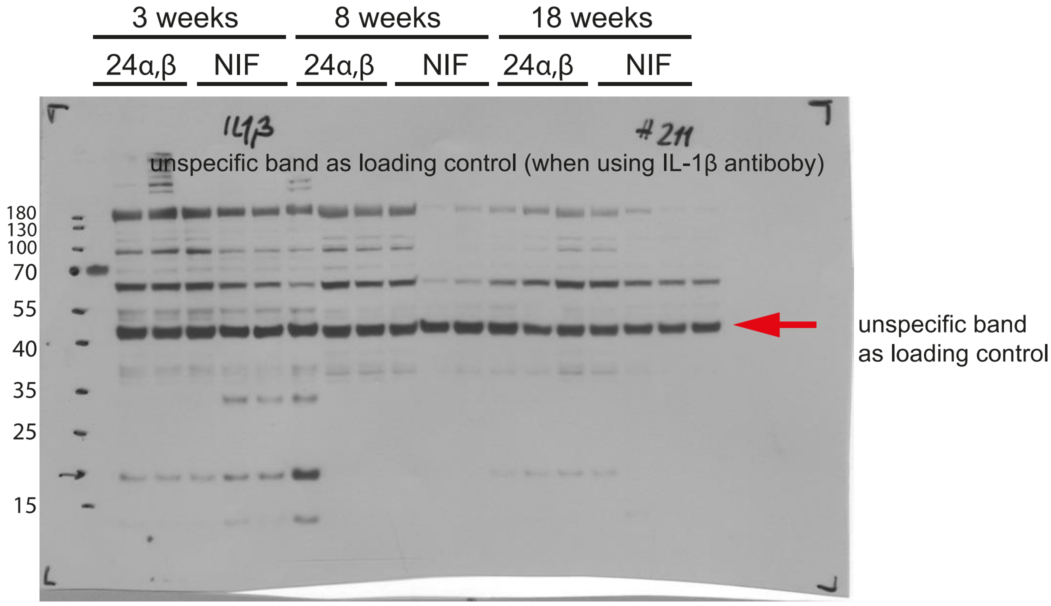

Liver supernatant samples:
Primary AB: anti-mouse Caspase-1 (Casper: 1:1000)
Secondary AB: Mouse TrueBlot ULTRA: anti-mouse Ig HRP (1:1000)

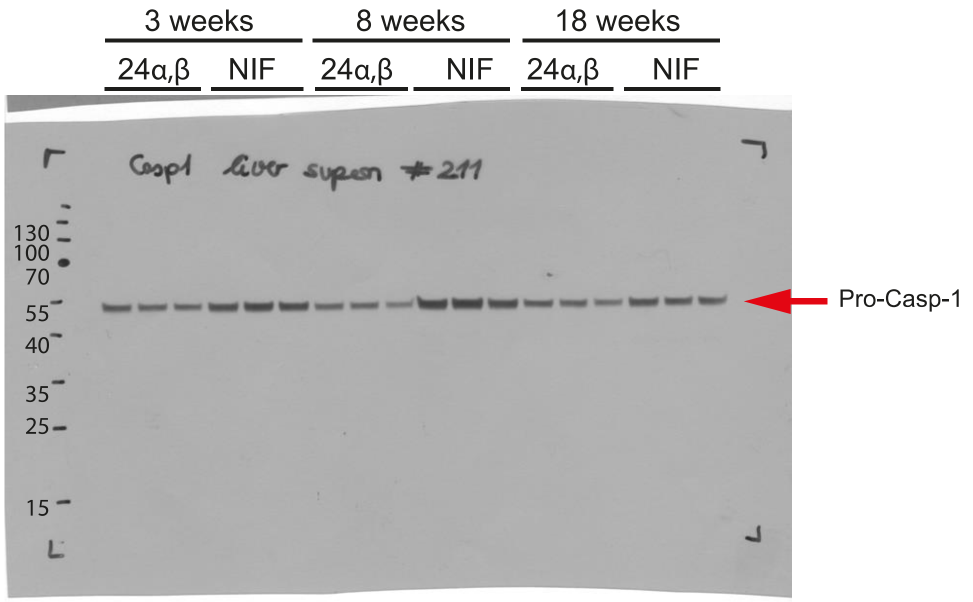

Long exposure

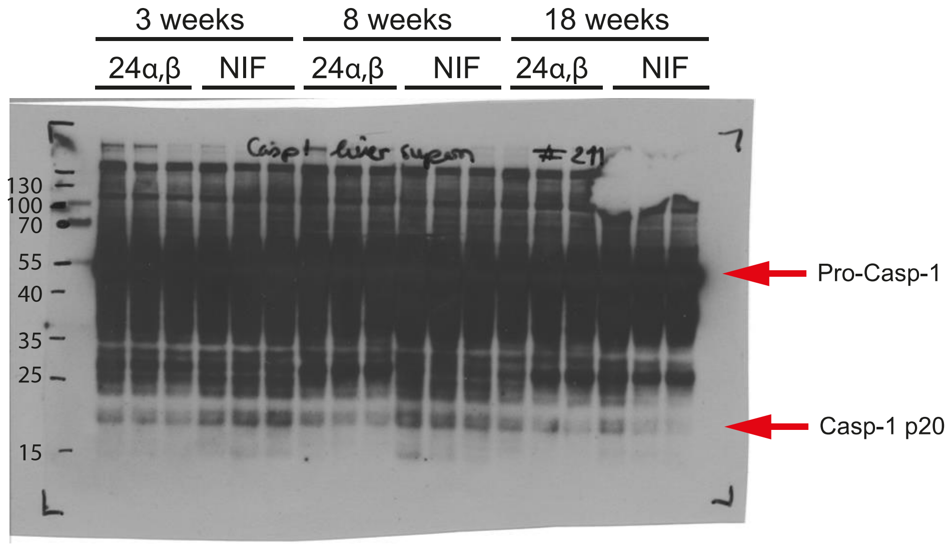

Liver supernatant samples:
Primary AB: anti-mouse IL-1β (1:2500)
Secondary AB: HRP-labelled anti-goat antibody (1:10000)

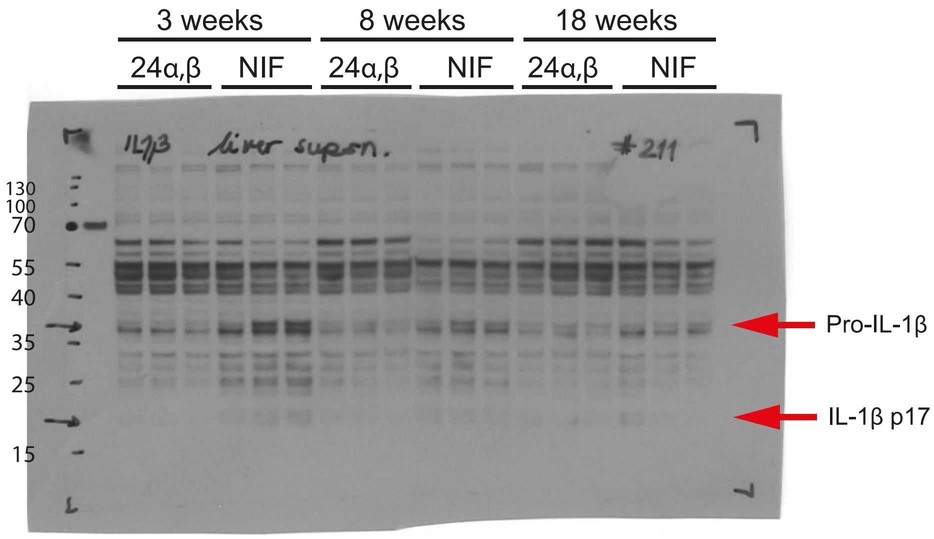

Long exposure

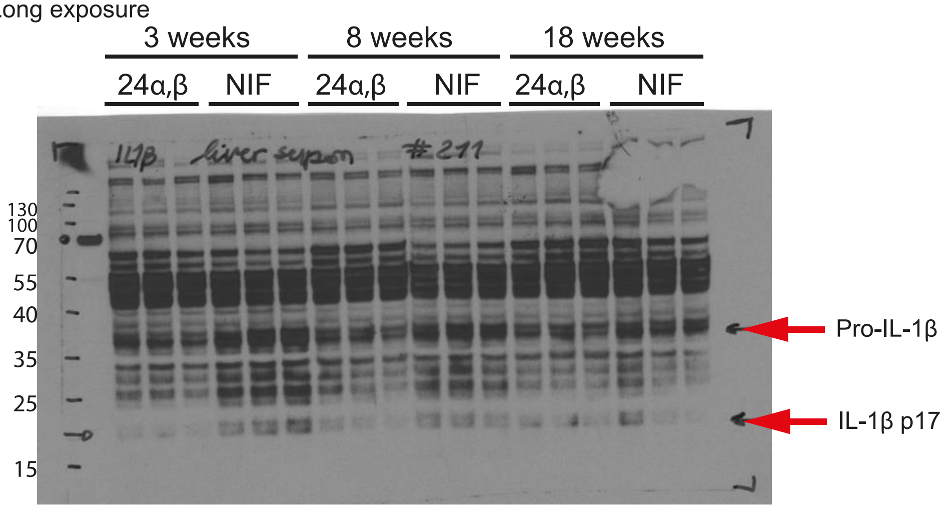

Fig S9. Related to Figures 4 and 5.

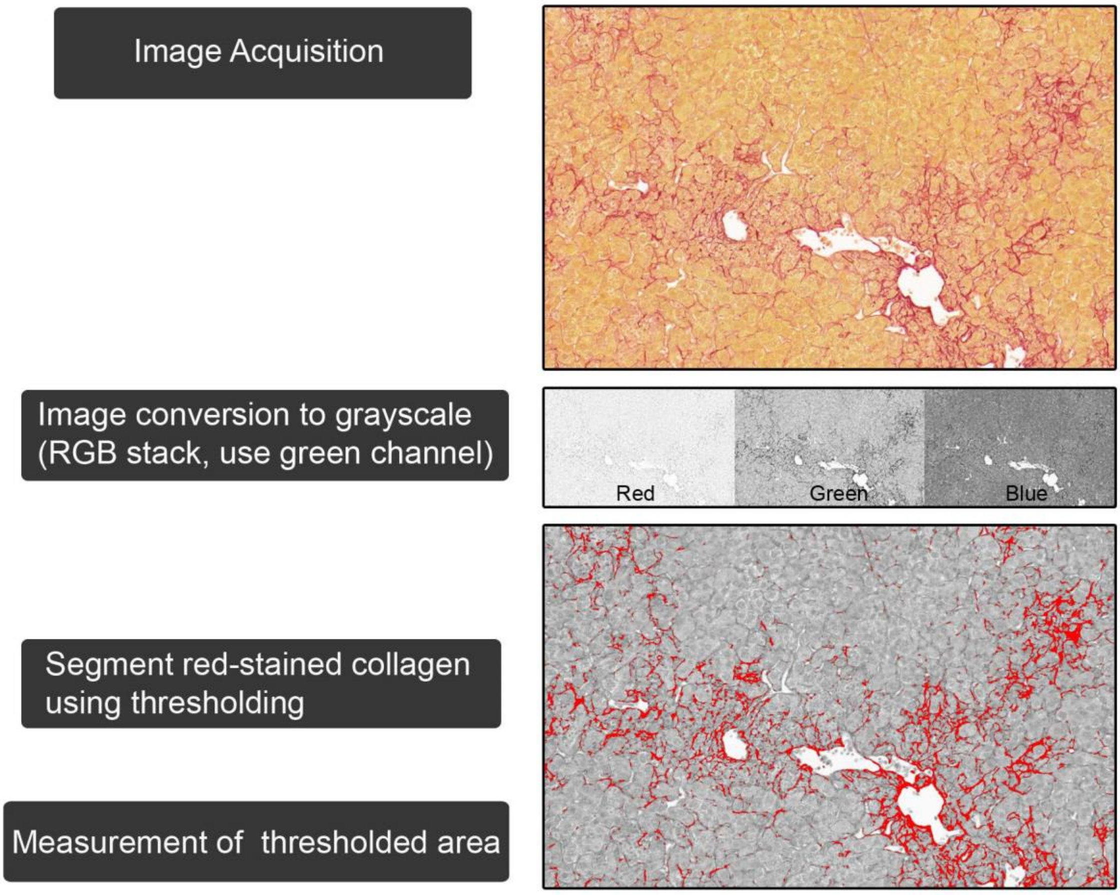

Fig S9. Quantification of PSR staining.
Outline of strategy for quantification of PSR stained liver sections in Image J. In a first step RGB images are converted into gray scale images (*Image>Type>RGB Stack* command) to split the image into red, green and blue channels. The green channel has the best separation and is used for thresholding the collagen signal (*Image>Adjust>Threshold* command). Within the "Threshold" tool the green channel is automatically thresholded and has to be manually adjusted. The threshold level arrived at manually is about half the automatically set level (in mean). The fact, that setting a threshold that is a fraction of the automatically determined threshold was used to create a macro that does not require manual thresholding. "Area Fraction" in per cent (limited to Threshold) was measured (*Analyse>Measure* command).

Supplementary Table 1. List of reagents and resources used in this study

Reagent or resource	Source	Catalog number	Dilution
**Antibodies for treatment**			
Anti-TGFβ, clone 1D11.16.8	BioXcell (Nordic biosite)	BP0057-25MG	
**Primary antibodies for IHC**			
goat anti-IL33	RnD	AF3626-SP	1:50
Rat anti-CD45	Abcam	ab25386	1:100
rabbit anti-ASMA	Abcam	ab5694	1:150
Rat anti-Pdgfrα	Abcam	ab51875	1:100
Rabbit anti-collagen I	Abcam	ab21286	1:200
**Secondary antibodies for IHC**			
Donkey α-goat Alexa 594	Invitrogen	A11058	1:500
Donkey α-rat Alexa 488	Invitrogen	A21208	1:1000
Donkey α-rat Alexa 594	Invitrogen	A21209	1:1000
Goat α-rabbit Alexa 488	Invitrogen	A11008	1:500
Goat α-rabbit Alexa 647	Invitrogen	A21245	1:1000
**Antibodies for flow cytometry**			
Vα3.2 (RR3-16)	BD Biosciences	553219	1:200
B220 (RA3-6B2)	BD Biosciences	553086	1:320
CD4 (RM4-5)	BD Biosciences	553045	1:640
CD19 (1D3)	BD Biosciences	563333	1:200
CD11b (M1/70)	BD Biosciences	563015	1:200
CD49b (DX5)	BD Biosciences	553856	1:200
Ly6G (1A8)	BD Biosciences	740953	1:200
CD8a (53-6.7)	BD Biosciences	553029	1:250
Siglec-F (E50-2440)	BD Biosciences	562681	1:150
CD49b (DX5)	eBioscience	12-5971-82	1:320
Ly-49G2 (4D11)	eBioscience	46-5781-82	1:200
CD8a (53-6.7)	eBioscience	25-0081-82	1:320
CD3ε (145-2C11)	eBioscience	17-0031-81	1:200
Vβ9 (MR10-2)	eBioscience	48-58-23-80	1:200

CD62L (MEL-14)	eBioscience	93-0621-42	1:200
CD19 (1D3)	eBioscience	13-0193-82	1:640
CD45 (30-F11)	Biolegend	103116	1:200
I-Ad (MHCII) (39-10-8)	Biolegend	115008	1:150
CD115 (AFS98)	Biolegend	135527	1:100
Ly6C (HK1.4)	Biolegend	128012	1:200
CD38 (90)	Biolegend	102717	1:200
F4/80 (BM8)	Biolegend	123116	1:100
CD3 (17A2)	Biolegend	100243	1:200
TER119 (TER-119)	Biolegend	116204	1:200
SAv-BV650	Biolegend	405231	1:400
Fixable viability dye	eBioscience	65-0866-14	1:200
**Antibodies for cytometric cell sorting**			
Vα3.2 (RR3-16)	eBioscience	17-5799-82	1:200
Vβ9 (MR10-2)	eBioscience	48-58-23-80	1:200
Propidium iodine	Sigma-Aldrich	P4170	1:500
**Antibodies for flow cytometry of CD2-GFP mice (alternative panel)**			
Vα3.2 (RR3-16)	BD Biosciences	553218	1:200
CD4 (PM4-5)	Biolegend	100555	1:200
CD8a (53-6.7)	BD Biosciences	551162	1:200
CD49b (DX5)	Biolegend	108921	1:200
CD3e (145-2C11)	eBioscience	17-0031-81	1:150
CD25 (PC61.5)	eBioscience	562284	1:200
CD45 (30-F11)	Biolegend	B185138	1:200
Vβ9 (MR10-2)	eBioscience	48-58-23-80	1:200
CD11b (M1/70)	BD Biosciences	563015	1:200
CD19 (1D3)	BD Biosciences	563333	1:200
PE-CF594 Streptavidin	BD Biosciences	562284	1:500
**Antibodies for Western blotting**			
goat anti-IL-1β	R&D Systems	AF-401-MA	1:2,500
Rabbit anti-NLRP3 (D4D8T)	Cell Signaling	15101	1:1,000
mouse anti-Caspase-1 (p20)	Adipogen	AG-20B-0042	1:1,000

HRP-labeled anti-rabbit Ig	Cell Signaling	7074	1:10,000
HRP-labeled anti-goat Ig	Santa Cruz	sc-2020	1:10,000
HRP-labeled anti-mouse Ig (eB144)	Rockland	18-8817-33	1:1,000
**Primers**	**Source**	**UniGene no.**	
SYBR green primers for *Tgfb1*	Qiagen	Mm.248380	
SYBR green primers for *Ctgf*	Qiagen	Mm.390287	
SYBR green primers for *Nlrp3*	Qiagen	Mm.54174	
SYBR green primers for *Nfkb*	Qiagen	NM_008689	
SYBR green primers for *Gapdh*	Qiagen	Mm.309092	
SYBR green primers for *Casp1*	Qiagen	NM_009807	
SYBR green primers for *Cxcl1*	Qiagen	NM_008176	
SYBR green primers for Ccl2	Qiagen	NM_011333	
SYBR green primers for Pdgfra	Qiagen	NM_001083316	
SYBR green primers for Il33	Qiagen	NM_001164724	
